# Lifetime Effects of Adherence to Cardiovascular and Diabetes Medications in Spain: A Modelling Study in a Population Cohort of 152,117 Patients

**DOI:** 10.1007/s40273-026-01597-1

**Published:** 2026-03-17

**Authors:** Alba Sánchez-Viñas, Runguo Wu, Ignacio Aznar-Lou, Borislava Mihaylova, Maria Rubio-Valera

**Affiliations:** 1 Health Technology Assessment in Primary Care and Mental Health (PRISMA) Research Group, Institut de Recerca Sant Joan de Déu, Santa Rosa 39-57, 08950 Esplugues de Llobregat, Spain; 2 Centro de Investigación Biomédica en Red de Epidemiología y Salud Pública (CIBERESP), Madrid, Spain; 3 Facultat de Medicina i Ciències de la Salut, Universitat de Barcelona, c. Casanova 143, 08036 Barcelona, Spain; 4 Health Economics and Policy Research Unit, Wolfson Institute of Population Health, Queen Mary University of London, Yvonne Carter Building, 58 Turner St, London, E1 2AB UK; 5 Health Economics Research Centre, Nuffield Department of Population Health, University of Oxford, Old Road Campus, Headington, Oxford, OX3 7LF UK; 6 Catalonian Cancer Strategy, Department of Health, Biomedical Research, Institute of Bellvitge L’Hospitalet de Llobregat (IDIBELL), Barcelona, Spain

## Abstract

**Objectives:**

Non-adherence to cardiovascular disease (CVD) treatments leads to suboptimal health outcomes and increased healthcare and societal costs. We assessed the long-term effects of adherence to CVD and diabetes medications using population data and microsimulation modelling.

**Methods:**

We developed a CVD microsimulation model using individual participant data from the SIDIAP database (2012–2021) for 152,117 adults who received new prescriptions for antihypertensive, lipid-lowering, oral glucose-lowering or antiplatelet treatments in Catalonia between January 2012 and December 2013. Model inputs included demographic and clinical characteristics, medication adherence and cardiovascular events. Costs (€, 2025) and treatment effects were sourced from the literature, and utilities were estimated using national population-based surveys. Model validity was assessed by comparing simulated and observed cumulative incidences over 8 years. The model simulated life-years (LYs), quality-adjusted life-years (QALYs) and healthcare and societal costs under three scenarios: non-adherent, observed adherence and full adherence. We estimated the maximum per-patient cost at which adherence-enhancing interventions would remain cost-effective.

**Results:**

Simulated cumulative incidences of cardiovascular events and all-cause death closely matched observed data. Improved adherence increased survival by 0.19–0.58 years and QALYs by 0.25–0.70, while increasing lifetime healthcare costs by €2,431–€8,093 per patient. The additional cost per QALY ranged from €8,946 to €12,614 per QALY, indicating that improving adherence is likely to be a cost-effective if achieved at additional cost of up to €4,041 to €10,098 per patient.

**Conclusions:**

Long-term extrapolation of real-world data using microsimulation modelling shows that optimising adherence to CVD and diabetes medications can enhance health outcomes cost-effectively.

**Supplementary Information:**

The online version contains supplementary material available at 10.1007/s40273-026-01597-1.

## Key Points for Decision-Makers

**Table:** 

By extrapolating real-world data using microsimulation modelling, this study estimates the lifetime health and costs consequences of improving adherence to cardiovascular disease and diabetes medications.
Across different adherence levels, higher adherence was associated with gains of 0.19–0.58 life-years and 0.25–0.70 quality-adjusted life-years (QALYs) per patient, alongside higher lifetime costs.
Despite the additional costs, ICERs ranged from €8,946 to €12,614 per QALY. Threshold analyses indicate that adherence-enhancing interventions would remain cost-effective at per-patient costs higher that those reported for existing interventions.

## Introduction

Despite advances in pharmacological therapies, cardiovascular disease (CVD) remains a leading global health challenge [1], imposing a high burden on individuals, healthcare systems and societies globally [2].

Diabetes, hypertension and dyslipidaemia are significant risk factors of CVD, contributing to the prevalence and severity of cardiovascular complications [3]. In addition, suboptimal medication adherence is a critical factor that hinders the effective management of CVD, increasing the risk of major cardiovascular events and death [4, 5].

Multiple factors influence this complex issue, including patient knowledge, beliefs, socioeconomic status and healthcare system dynamics [6]. Approximately 50% of patients fail to adhere to prescribed medication regimens for CVD [7], while adherence rates for diabetes treatments are slightly higher [8]. The lack of adherence leads to suboptimal health outcomes and increased healthcare utilisation, further exacerbating the overall burden of CVD [9–11].

Addressing the significant challenge of medication non-adherence in CVD requires the development and implementation of effective interventions [12] and demonstration of their long-term benefits. Decision analytic health economic models allow researchers to project long-term outcomes by analysing data over extended periods [13] and are useful for evaluating such interventions.

While several economic models have explored the cost-effectiveness of CVD treatments [14–16] most tend to assume full adherence, which overlooks the real-world impact of treatment non-adherence on patient outcomes and healthcare resource utilisation. Only a few models have specifically evaluated the effects of medication adherence [17, 18]. These studies highlight that poor adherence can substantially affect health outcomes and cost-effectiveness, exacerbating the economic burden and reducing quality of life (QoL) due to accelerated disease progression, underscoring the importance of integrating medication adherence into economic evaluations of CVD treatments.

Real-world data (RWD) from electronic healthcare records enables valuable studies of medication adherence [19]. Electronic health records capture ongoing patient care over extended periods, enabling tracking of long-term outcomes, adherence patterns and treatment outcomes beyond the typical duration of clinical trials [20]. When combined with clinical trial evidence, RWD can provide a more generalisable understanding of treatment effects across the population.

This study aims to develop a microsimulation model on the basis of RWD to assess the lifetime impact of CVD and diabetes treatments on health outcomes and costs and provide insights into how adherence influences treatment outcomes and healthcare costs. We assess the effects of medications under key adherence scenarios, which allows us to analyse the cost-effectiveness of treatments depending on level of adherence.

## Methods

### Study Population

We developed a CVD Markov model using the longitudinal register-based anonymous and encoded RWD from the SIDIAP database. This database contains anonymised electronic health records from the Catalan Institute of Health, the main public healthcare provider in Catalonia, covering 76% of the population at the primary care level [21].

The study cohort encompassed men and women aged 18 and older, who were issued a new prescription for antihypertensive, lipid-lowering, oral glucose-lowering or antiplatelet treatments in the public primary care in Catalonia, Spain, between 1 January 2012, and 31 December 2013. Primary care is the initial access point to the public healthcare system and acts as a gatekeeper for secondary care services. General practitioners issue a substantial number of prescriptions, which are subsequently dispensed by community pharmacists. Notably, participants in this study could receive multiple new prescriptions during the observation period. The pharmacotherapeutic groups of the medications included in the study and the details of excluded medications are outlined in Supplementary Table ST1. To focus on longer-term medication use, the study did not consider prescriptions with a duration of use under 100 days.

The index date, marking each patient’s entry into the cohort, was defined as the first time a patient received a new prescription. A prescription was considered new if there had been no other prescription from the same pharmacological subgroup (3rd level of the Anatomical Therapeutic Chemical classification) in the 6 months preceding the index date.

A minimum follow-up of 1 year in the database was required for inclusion in the study. The observation period ended on the date of death, loss to follow-up or 30 June 2021, with an observation period limited to 8 years.

Baseline characteristics, including sex, age, socioeconomic deprivation (Supplementary Methods A), smoking history and CVD, were recorded. Information on CVD included cardiovascular risk factors (hypertension, diabetes, dyslipidaemia and atherosclerosis) and history of cardiovascular events (stroke, coronary heart disease [CHD] and heart failure [HF]). Diagnoses were categorised on the basis of the International Classification of Diseases, 10th Revision (ICD-10), and validated through clinical criteria defined by practising physicians (Supplementary Table ST2). Dates of occurrence of stroke, CHD and HF, along with prescriptions and dispensing of CVD and diabetes treatments during follow-up, were recorded in the database.

### CVD Microsimulation Model

**Fig. 1:**
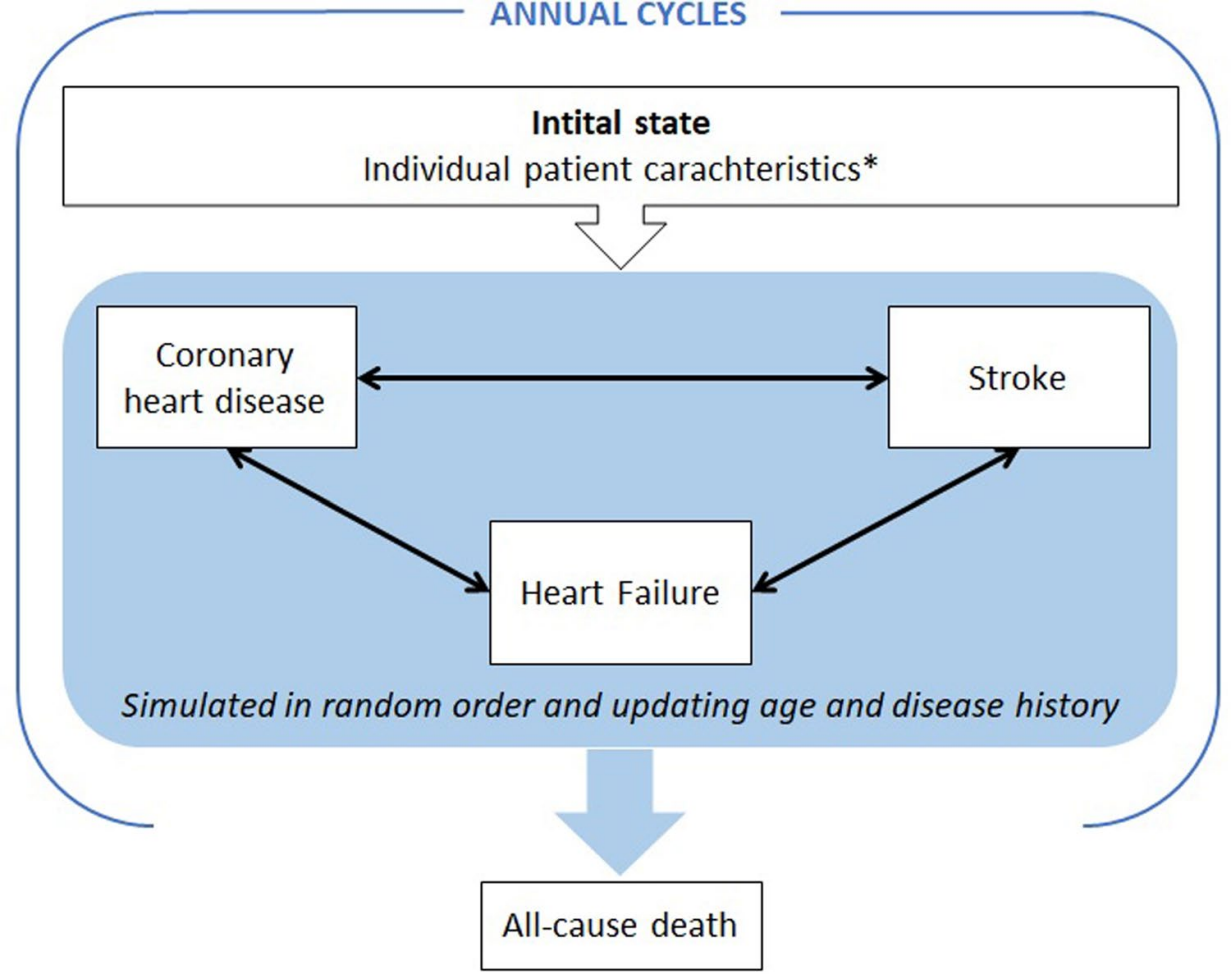
Schematic representation of the cardiovascular disease Markov model. The initial state represents the first time a patient receives a new prescription for oral glucose-lowering, lipid-lowering, antihypertensive or antiplatelet treatment, which can be prescribed for primary or secondary prevention. Patients remain in the initial state until the first cardiovascular event. *Individual patient characteristics at baseline include: sex; socioeconomic deprivation; age and smoking status at the time of the index prescription; history of stroke, heart failure, or coronary heart disease and diagnoses of diabetes, dyslipidaemia, hypertension or atherosclerosis prior to the index prescription

Figure 1 presents a schematic of the state-transition CVD model, which is based on predicting the first occurrence of four key events: stroke, CHD, HF and all-cause death. To determine the transition probabilities between model states, we estimated four risk equations using the SIDIAP database (one for each key event) on the basis of parametric survival regression models with time-updated age, new event history and treatments, and fixed other patient characteristics at baseline (Supplementary Table ST3). Of the variables available in the database, sociodemographic (age, sex and socioeconomic deprivation) and clinical characteristics (CVD and smoking history, cardiovascular events during follow-up and medication adherence) at the index date were selected as model covariates on the basis of external evidence [3] and expert opinion regarding their relevance to cardiovascular risk. Age was centred in the analyses to facilitate interpretation of regression coefficients and improve stability of the regression models. Specifically, age was centred at 55 years old, which represents the average of the mean age of the study cohort and the mean age of the Catalan Health Survey population. The estimated risk equations were used to predict the risk of the four key events during each cycle in the model.

The model simulates the transition of patients from the initial state at the time of the index prescription to the key events using an annual model cycle and lifetime horizon (until all patients die or reach 100 years of age) [22]. Mortality risk includes short- and long-term effects of cardiovascular events, distinguishing risk of death in year of event and risk of death in following years. The model predicts the annual occurrence of stroke, CHD, HF and all-cause mortality, lifetime health and healthcare and societal costs. Life years (LY), quality-adjusted life-years (QALYs) and costs were discounted at an annual rate of 3% in the base case analysis. The model was built using Python 3.8 within Visual Studio Code. The full code is openly available at https://zenodo.org/records/17372944, ensuring transparency and reproducibility [23].

More detailed information on the modelling procedure is available in Supplementary Methods B.

### Medication Adherence

Real-world patient medication adherence was directly assessed using the longitudinal SIDIAP population dataset. For each pharmacotherapeutic group, adherence was calculated annually as the proportion of days covered (PDC), and reporting followed the TEN-SPIDERS framework [24]; details are provided in Supplementary Table ST4. Dispensed medication boxes were converted to days’ supply and mapped on a day-level timeline using dispensing dates, allowing carry-over of overlapping supplies within the same pharmacotherapeutic group. Annual PDC was then calculated as the number of covered days divided by the number of days in the year, and patients were deemed adherent if they had a PDC of ≥ 80% [25], equivalent to a minimum of 10 medication boxes dispensed annually.

To reflect real-world adherence patterns in the model, observed adherence was incorporated starting from the cycle in which the prescription was initiated and was used for the initial stages of each patient’s simulation until the end of the follow-up period. Subsequently, the last observation carried forward method was implemented, whereby the patient’s last recorded adherence was carried forward until the final cycle.

### Medication Adherence Scenarios

Three adherence scenarios: observed adherence, full adherence and no treatment (non-adherence) were incrementally compared. Observed adherence represents the adherence levels during follow-up in the study cohort. In the full adherence scenario, all patients were assumed to maintain optimal adherence (PDC ≥ 80%) throughout the entire time horizon. The non-adherent scenario represents a counterfactual condition in which all individuals are modelled as non-adherent to prescribed therapies for the full simulation.

The CVD model was used to project lifetime outcomes and costs associated with each adherence scenario. The incremental cost per life year gained and per QALY gained were evaluated from both healthcare and limited societal perspectives.

### Treatment Effects

The model simulates the effects of treatments on the risk of major cardiovascular events and mortality, using relative risks derived from meta-analyses of randomised controlled trials, as detailed in Supplementary Table ST5 [26–31].

Survival equations were estimated from a partially treated real-world population. To avoid double-counting treatment effects, we first established a truly untreated baseline before applying relative treatment effects from the literature. For each patient and cycle, the linear predictor was adjusted to remove the treatment effect already implicit in the data, calculated as the product of the patient’s observed adherence and the literature-based relative risk (RR) for each pharmacotherapeutic group (ADH × ln(RR)). From this untreated baseline, treatment effects from the literature were re-applied according to the corresponding adherence scenario. In the observed-adherence scenario, treatment effects were applied on the basis of each patient’s observed adherence level (PDC ≥ 80%) during the corresponding annual period. In the full-adherence scenario, adherence was assumed to be complete (ADH = 1) for all treatments from the index date to the last cycle. In the non-adherent scenario, no treatment effects were applied.

### Health-Related Quality of Life (QoL)

The Catalan Health Survey [32] regularly assesses health outcomes, including quality of life on the basis of the EuroQoL 5-dimensions 5-level questionnaire (EQ-5D-5L), on a representative sample of the Catalan population (annual *N *≈ 5000). Data on the EQ-5D-5L for years 2019–2022 was used to estimate utilities on the basis of Spanish tariffs for the different states (except heart failure) using a linear regression model adjusted by demographic and clinical variables (see Supplementary Methods C). The sample characteristics of the Catalan Health Survey dataset are available in the Table SMC1. Coefficients from this model were integrated into the CVD model to annually estimate QoL (Table SMC2).

Since the survey did not record the incidence of heart failure, QoL utility of heart failure was derived from the literature [33] and adapted for the study cohort’s demographic characteristics and comorbidities resulting in an estimated mean QoL reduction of 0.184 for heart failure.

QoL utilities in each model state were combined with survival data to estimate QALY.

### Missing Data

Missing data ranged from 12% (socioeconomic deprivation) to 16% (smoking status) in the SIDIAP database and from 0.12 to 5.55% in certain variables (diabetes, dyslipidaemia, hypertension, socioeconomic status and smoking status) in the Catalan Health Survey [32] database. Multiple imputation by chained equations, employing predictive mean matching with the five nearest neighbours across ten imputed datasets for all characteristics, was implemented. Estimated risk and QoL regression models on imputed datasets were combined following Rubin’s rule [34]. Further information about missing data handling in the study can be found in Supplementary Methods D.

### Costs

We conducted a cost analysis from two distinct perspectives: the healthcare perspective and the limited societal perspective [22] each capturing different cost components associated with managing cardiovascular conditions. Costs were inflated to 2025 in Spain [35] where necessary.

**Healthcare Perspective**: This perspective included direct medical costs for prescribed treatments and cardiovascular events. Annual treatment-related costs included medication expenses and the costs associated with regular medical consultations and tests, as recommended by the clinical guidelines of the Catalan Healthcare system [36–38]. Healthcare resource costs were obtained from the tariffs published in the Official Government Bulletin [39]. Medication costs were based on the prices of generic medications. Direct medical costs associated with cardiovascular events were sourced from the literature [40–43]. These costs were added in the year of the event and subsequent years.

**Limited Societal Perspective:** In addition to healthcare costs, this perspective considered costs such as productivity losses due to work morbidity-induced absenteeism, informal care and lost productivity resulting from premature mortality as a consequence of CVD. Indirect costs of cardiovascular events (i.e. cost of work morbidity-induced productivity loss and costs of informal care) were obtained from the literature [40–42]. Productivity losses due to premature mortality were calculated by multiplying the days of absence from work by the mean annual salary in Spain for 2023 [44], and adjusted using the activity rate in Spain in the 4th quarter of 2023 [45].

Further details regarding unit costs, inflation adjustment, and other relevant information can be found in Supplementary Methods E.

To explore the role of cost of implementing adherence-enhancing interventions, a threshold analysis estimated the maximum intervention cost per patient at which an intervention remains cost-effective at a €25,000/QALY threshold.

### Model Validation

To check the accuracy of the CVD model, we conducted a comparative analysis of the observed and predicted cardiovascular events in the study cohort over 8 years using the Nelson–Aalen cumulative hazard function, and the model-simulated cardiovascular event rates for the initial eight cycles.

### Sensitivity Analysis

Sensitivity analyses were conducted to examine the impact of discounting on the study results, with both outcomes and costs presented undiscounted or discounted at 5% per annum following local guidelines [22].

### Uncertainty Analysis

A bootstrap approach was used to assess uncertainty in the model. For computational feasibility, this involved drawing 500 bootstrap samples with replacement from the study cohort and re-estimating the risk models. Furthermore, 500 bootstraps with replacement samples were drawn from the Catalan Health Survey dataset to address uncertainty in QoL estimates.

Monte Carlo techniques were employed to handle uncertainty around treatment effects and CVD costs. This involved drawing values for these parameters from their distributions (see Supplementary Table ST6 for further details).

Incremental costs per LY and QALY’s gained were estimated for each bootstrap sample to quantify the economic impact of the different intervention strategies.

Stratum-specific analyses were conducted across pharmacological groups, sociodemographic characteristics and primary versus secondary prevention categories to further explore variability in cost-effectiveness and utility outcomes.

A completed CHEERS checklist [46] is provided in the supplementary materials.

## Results

### Baseline Characteristics of the Study Population

Between January 2012 and December 2013, 152,117 patients in Catalonia received new prescriptions for selected treatments in primary care. Antihypertensive drugs were the most frequently prescribed treatment (59%), followed by lipid-lowering drugs (42%), antiplatelet drugs (14%) and oral glucose-lowering drugs (11%), with approximately 6% of these treatments prescribed for secondary CVD prevention.

Overall, the cohort was evenly split by sex and had a mean age of 57 years. However, patients receiving antiplatelet and oral glucose-lowering medications were predominantly males and patients receiving antiplatelet treatments were older (mean age 64 years). More than 60% of population was categorised with intermediate or low-intermediate socioeconomic deprivation status, with a comparable distribution observed across different pharmacotherapeutic groups. At the time of prescription, the most common diagnoses were high blood pressure and high cholesterol. More than half of the patients (53%) reported having never smoked (Table 1).

**Table 1:** Baseline characteristics of patients in the study cohort, by medication subgroup of index prescription and overall

	Cohort by medication subgroup				Overall*
	Antihypertensive treatment	Oral glucose-lowering treatment	Lipid-lowering treatment	Antiplatelet treatment	
*N* (%)	90,485 (59.48)	17,373 (11.48)	63,207 (41.55)	20,675 (13.59)	152,117 (100)
Age^a^ – Mean (SD)	58.56 (14.92)	57.33 (13.99)	56.44 (12.68)	64.41 (15.57)	57.15 (14.29)
*Age*^*a*^* (%)*					
≤ 46 years old	20,356 (22.50)	3996 (22.98)	14,119 (22.34)	2814 (13.61)	36,642 (24.09)
47–55 years old	21,388 (23.64)	4448 (25.60)	17,775 (28.12)	3556 (17.20)	38,653 (25.41)
56–65 years old	21,793 (24.08)	4500 (25.90)	17,897 (28.31)	4932 (23.85)	38,152 (25.08)
66–76 years old	14,894 (16.46)	2627 (15.12)	9,111 (14.41)	4219 (20.41)	22,987 (15.11)
≥ 77 years old	12,054 (13.32)	1805 (10.39)	4,305 (6.81)	5154 (24.93)	15,683 (10.31)
*Sex (%)*					
Female	44,197 (48.84)	6799 (39.14)	29,313 (46.38)	8561 (41.41)	73,661 (48.42)
Male	46,288 (51.16)	10,574 (60.86)	33,894 (53.62)	12,114 (58.59)	78,456 (51.58)
*Cardiovascular risk factors*^*a,b*^* (%)*					
Diabetes	7937 (8.77)	17,373 (100)	7522 (11.91)	2865 (13.86)	21,017 (13.82)
Dyslipidaemia	25,145 (27.79)	7429 (42.76)	63,207 (100)	11,017 (53.29)	73,580 (48.37)
Hypertension	90,485 (100)	6638 (38.21)	19,481 (30.82)	12,014 (58.11)	94,493 (62.12)
Atherosclerosis	700 (0.77)	172 (0.99)	821 (1.30)	1152 (5.57)	1618 (1.06)
*History of cardiovascular events*^*a*^* (%)*					
Stroke	2405 (2.66)	417 (2.40)	2423 (3.83)	3401 (16.45)	4498 (2.96)
Heart failure	1271 (1.40)	173 (1.00)	362 (0.57)	478 (2.31)	1314 (0.86)
Coronary heart disease	2576 (2.85)	276 (1.59)	2415 (3.82)	2631 (12.73)	2930 (1.93)
*Socioeconomic deprivation*^*a,c,d*^* (%)*					
Low	11,740 (12.97)	1880 (10.82)	8559 (13.54)	3086 (14.93)	19,389 (12.75
Low intermediate	27,310 (30.18)	4796 (27.61)	19,764 (31.27)	6261 (30.28)	46,119 (30.32)
Intermediate	29,471 (32.57)	5677 (32.68)	20,201 (31.96)	6640 (32.12)	49,643 (32.63)
High intermediate	17,681 (19.54)	3881 (22.34)	11,874 (18.79)	3760 (18.19)	29,647 (19.49)
High	4283 (4.73)	1139 (6.56)	2809 (4.44)	928 (4.49)	7319 (4.81)
*Smoking history*^*a,d*^* (%)*					
Non-smoker	50,189 (55.47)	8968 (51.62)	30,877 (48.85)	9956 (48.15)	81,078 (53.30)
Smoker	24,066 (26.60)	5124 (29.49)	21,299 (33.70)	6634 (32.09)	44,108 (29.00)
Ex-smoker	16,230 (17.94)	3281 (18.89)	11,031 (17.45)	4085 (19.76)	26,931 (17.70)

*Patients can have more than one treatment prescribed

^a^ Information at the time of the index prescription

^b^4.030 patients (2.65%) did not have a diagnosis of cardiovascular disease or diabetes (ICD-10 codes E00-E90 or I00-I99) registered at the time of the index prescription

^c^Deprivation Index 2011 of the Spanish Society of Epidemiology (IP2011): combines six socioeconomic indicators (percentage of manual working population, casual working population, unemployed population, population with insufficient education, young population with insufficient education and homes without Internet access) for each census section, calculated from the 2011 Spanish Population and Housing Census data (Duque I, Domínguez-Berjón MF, Cebrecos A, et al. Índice de privación en España por sección censal en 2011. *Gac Sanit*. 2021;35(2):113–122. 10.1016/j.gaceta.2019.10.008). Detailed information in Supplementary Methods A

^d^Missing data imputed using multiple imputation by chained equations (MICE)

During the 8-year follow-up period, the proportion of patients who were prescribed additional medications varied depending on the treatment type. The use of concomitant medication ranged from 7 to 55% of patients, as detailed in Supplementary Table ST7.

**Fig. 2:**
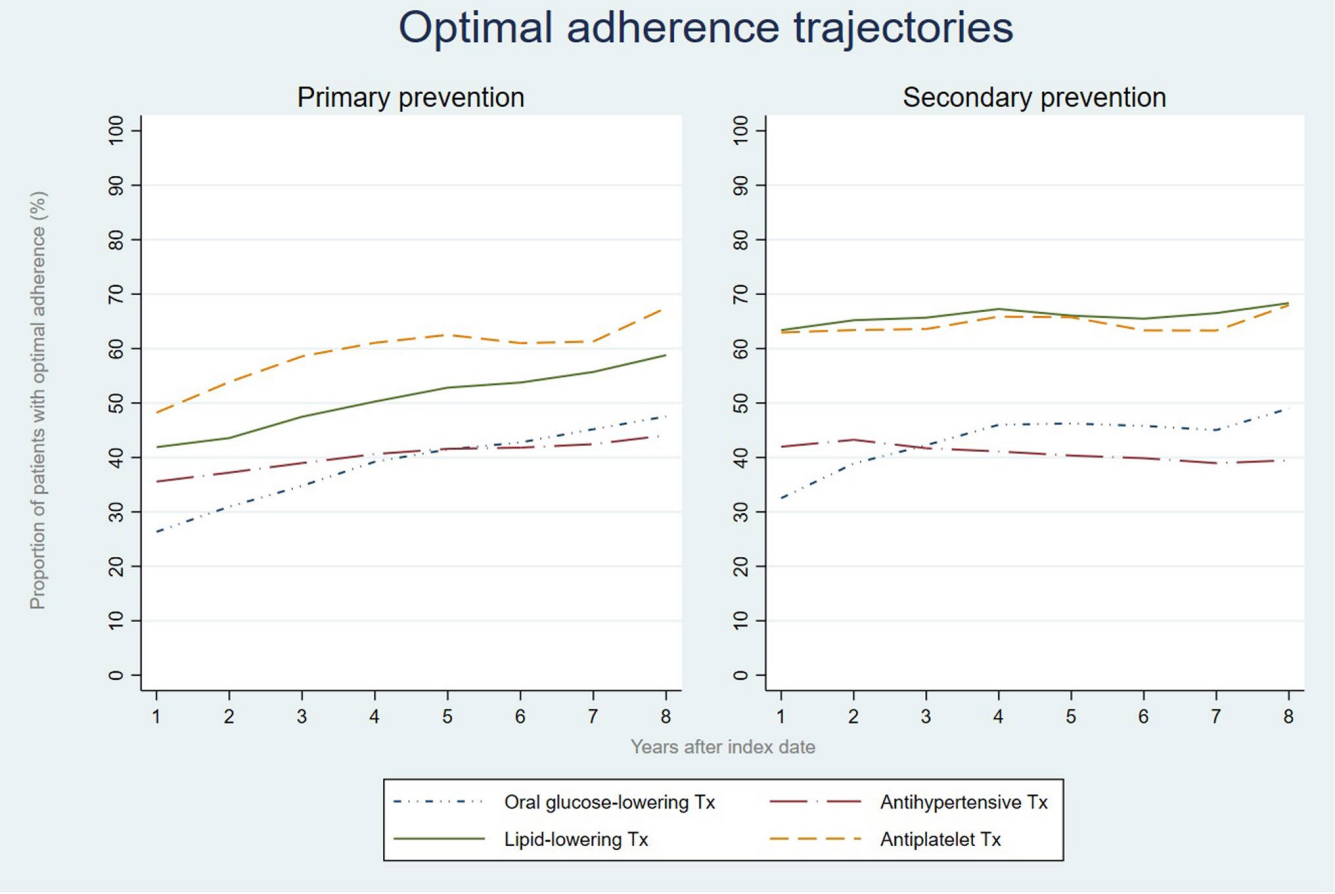
Adherence patterns for patients with optimal adherence in the study cohort for each medication subgroup of index prescriptions. NOTE: The index date ranges from 1/1/2012 to 31/12/2013. *Only observed data are represented in the figure. For the cohort of 152,117 patients, the proportion of administratively censored and dead patients increases annually for each treatment: Antihypertensive treatments (year 2 = 1.90%, year 8 = 25.78%); oral glucose-lowering medications (year 2 = 1.65%, year 8 = 26.06%); lipid-lowering medications (year 2 = 0.88%, year 8 = 21.15%); antiplatelet medications (year 2 = 3.69%, year 8 = 34.50%). Optimal adherence is defined as an annual proportion of days covered (PDC) ≥ 80%

Figure 2 shows adherence patterns throughout follow-up. Patients on secondary prevention were more adherent than those on primary prevention. For all treatments, adherence increased over the years for primary prevention, and antiplatelet therapy showed the highest proportion of patients adhering throughout the follow-up period. Adherence to other treatments was similar for primary and secondary prevention, except for lipid-lowering drugs, which showed higher adherence in secondary prevention. No differences in adherence patterns were observed between sexes or socioeconomic deprivation groups. However, younger patients exhibited lower levels of adherence than older patients, except for those on oral glucose-lowering treatment (see Supplementary Fig. SF1).

### Model Validation

In the internal model validation, the model reproduced the expected ordering across scenarios, with higher cumulative incidence in the non-adherent scenario and close alignment between the observed-adherence and observed rates (see Supplementary Fig. SF2).

### Projected Outcomes of Optimising Adherence

The model predicted outcomes and costs for each adherence scenario are presented in Table 2. For the overall cohort, as well as separately for primary and secondary CVD prevention patients, LY, QALY and costs increase with adherence.

**Table 2:** Predicted outcomes and costs over a lifetime for non-adherent patients and patients at different levels of medication adherence in primary, secondary prevention and overall

	Non-adherence		Observed adherence		Full adherence	
	Mean ^a^	95% CI ^b^	Mean ^a^	95% CI ^b^	Mean ^a^	95% CI ^b^
**Life years (LY) per person**						
Primary prevention	17.63		17.81		18.19	
Secondary prevention	11.95		12.44		12.93	
Overall*	*17.32*	*(17.28; 17.30)*	*17.51*	*(17.47;17.50)*	*17.90*	*(17.86;17.89)*
**Quality Adjusted Life Years (QALY) per person**						
Primary prevention	13.94		14.17		14.61	
Secondary prevention	5.24		5.95		6.52	
Overall*	*13.46*	*(13.38; 13.41)*	*13.71*	*(13.64; 13.67)*	*14.16*	*(14.10; 14.12)*
**Lifetime healthcare costs (€) per person**						
Primary prevention	2932		5269		11,060	
Secondary prevention	42,900		46,936		50,423	
Overall*	*5157*	*(5,150; 5,181)*	*7588*	*(7,583; 7,613)*	*13,250*	*(13,253; 13,282)*
**Lifetime healthcare + societal costs (€) per person**						
Primary prevention	5282		7480		13,022	
Secondary prevention	83,536		87,314		90,593	
Overall*	*9638*	*(9593; 9668)*	*11,923*	*(11,884; 11,956)*	*17,340*	*(17,311; 17,380)*

All discounted at 3% per annum

Primary prevention *N* = 143,651; secondary prevention *N* = 8,466

*All prescribed treatments considered

^a^Mean of the deterministic analysis

^b^95% CI of 500 bootstraps from sensitivity analysis

The predicted outcomes and costs stratified by treatment, sex and age, were consistent with the main findings: both outcomes and costs increased with adherence across all subgroups (Supplementary Fig. SF3). As expected, more LYs and QALYs were projected for patients of a younger age and secondary CVD prevention patients were projected to incur higher costs compared with primary prevention.

Table 3 presents the cost-effectiveness results from both the healthcare and limited societal perspectives. Overall, incremental costs per life year and per QALY gained were lower when adopting the limited societal perspective. Incremental costs per QALY and LYs gained were higher in primary prevention compared with secondary prevention. In primary prevention, the incremental costs per QALY gained ranged from €9,627 to €13,094 and incremental costs per LY gained from €12,666 to €15,230. In contrast, secondary prevention showed lower values, with the incremental costs per QALY gained between €5,268 and €6,204 and incremental costs per LY gained between €6,674 and €8,288.

**Table 3:** Incremental cost per life year and incremental cost per QALY, discounted at 3% across three scenarios for patients in primary and secondary prevention and the overall cohort

	Observed adherence versus non-adherence (ref.)	Full adherence versus observed adherence (ref.)	Full adherence versus non-adherence (ref.)
	Mean^a^	Mean ^a^	Mean ^a^
***Healthcare perspective***			
*Incremental cost per life year gained (€/LY gained)*			
Primary prevention	13,464	15,230	14,677
Secondary prevention	8288	7098	7691
Overall*	*12,729*	*14,655*	*14,018*
*Incremental cost per QALY gained (€/QALY gained)*			
Primary prevention	10,234	13,094	12,121
Secondary prevention	5627	6204	5881
Overall*	*9514*	*12,614*	*11,490*
***Limited societal perspective***			
*Incremental cost per life year gained (€/LY gained)*			
Primary prevention	12,666	14,574	13,976
Secondary prevention	7760	6674	7215
Overall*	*11,970*	*14,015*	*13,339*
*Incremental cost per QALY gained (€/QALY gained)*			
Primary prevention	9627	12,531	11,542
Secondary prevention	5268	5834	5516
Overall*	*8946*	*12,064*	*10,933*

All discounted at 3% per annum

Primary prevention *N* = 143,651; secondary prevention *N* = 8466.

*All prescribed treatments considered

^a^Mean of the deterministic analysis

Supplementary Fig. SF4 presents incremental costs per life year and per QALY gained estimates across pharmacotherapeutic groups, stratified by age, sex and prevention level. Improving adherence was generally cost-effective across all treatments, with most values below €20,000 per QALY or LY gained, especially among males and those patients in secondary prevention.

Depending on the scenario and cost perspective, the threshold intervention cost at which an adherence enhancing intervention remains cost-effective ranged between €4,041 and €4,184 for observed adherence versus non-adherence, €9,711 and €10,098 for full adherence versus non-adherence, and €5,411 and €5,914 for full versus observed adherence (Supplementary Table ST8).

### Sensitivity Analysis

Analyses using different discount rates were consistent with the base-case results, as costs and outcomes do not change significantly (Supplementary Table ST9).

### Uncertainty Analysis

The probabilistic sensitivity analysis (PSA) showed that all incremental costs per life year and per QALY gained consistently fell within the northeast quadrant of the cost-effectiveness plane, indicating that the improved adherence is both more effective and more costly (Supplementary Fig. SF5). Across all scenarios’, improving adherence was 100% cost-effective at a willingness-to-pay threshold between €10,000 and €20,000 per QALY (Supplementary Fig. SF6). Across 100–500 bootstrap replications, mean incremental costs per LY and QALY gained estimates were consistent, with narrow 95% confidence intervals indicating stable uncertainty estimates (Supplementary Fig. SF7).

## Discussion

The findings of this study underscore that improvements in adherence to CVD and diabetes medications can lead to meaningful gains in life expectancy and QALYs, while remaining cost-effective across different scenarios.

This study involved a novel methodological approach, which involved using microsimulation techniques informed by electronic population health records to simulate individual behaviour and outcomes. This allowed us to capture both patient-level heterogeneity and population-wide patterns in treatment initiation, adherence and cardiovascular outcomes. By leveraging individual patient data, the model reflects the complexity of real-world clinical practice while remaining generalisable to the broader target population.

The observed adherence rates in our cohort were consistent with previous reports of suboptimal adherence to CVD medications [4]. We observed variation in adherence patterns by age and disease severity: older patients and those receiving secondary prevention exhibited higher adherence rates. This may be attributed to their elevated baseline cardiovascular risk [47], which could increase motivation to follow treatment recommendations.

Unlike previous studies relying on average adherence estimates [13], our analysis used individual adherence trajectories derived from electronic health records. This allowed for a more accurate representation of medication use and its effect on clinical and economic outcomes. Comparing scenarios of no treatment, observed adherence and full adherence highlighted the contrast between real-world behaviour and ideal clinical conditions. As expected, assuming full adherence overestimates health benefits and underestimates costs, owing to improved cardiovascular control under optimal conditions [48]. By explicitly incorporating adherence into the cost-effectiveness framework, we provided a more realistic estimation of long-term outcomes and resource use, underscoring the importance of using real-world adherence data in health economic evaluations.

The incremental costs per life year and per QALY gained across different adherence scenarios, were mostly below Spain’s €25,000 willingness-to-pay threshold [49]. These findings align with existing literature suggesting that adherence-enhancing interventions could be cost-effective [50, 51]. However, the incremental costs per life year and per QALY gained reported here represent lower-bound estimates of cost-effectiveness, as they do not account for the costs of specific interventions to increase adherence. In the context of CVD, strategies including educational programmes and reminder systems can effectively improve medication adherence, patient engagement and health outcomes [52, 53], yet their real-world effectiveness is often modest and inconsistent [54].

A threshold analysis was conducted to estimate the maximum per-patient cost at which an adherence-enhancing intervention would remain cost-effective, assuming a willingness-to-pay threshold of €25,000 per QALY. Depending on the scenario and cost perspective, adherence interventions could remain cost-effective with marginal costs of up to approximately €4,041–€10,098 per patient. These values are substantially higher than the intervention costs reported in the literature. For example, Chapman et al. [55] reported per-patient intervention costs ranging from $10–$142/patient for cardiovascular medication adherence programmes, depending on intervention intensity, while more recent evidence from pharmacist-led adherence interventions suggests annual implementation costs in the range of $246–$292/patient [56]. However, their actual cost-effectiveness would depend on achieving the level of adherence improvement assumed in our scenarios, which may be challenging in real-world settings.

A key strength of this study is its ability to simulate the effects of medication adherence at the individual level, providing a detailed assessment of how intermediate factors such as adherence influence long-term outcomes, such as cardiovascular events and mortality. This was made possible by leveraging comprehensive electronic health records from the SIDIAP database, which provide rich clinical information and long-term follow-up, thereby enhancing both the validity and generalisability of the model. Furthermore, the use of quality-of-life estimates derived from the Catalan Health Survey ensures that utility values are contextually appropriate for the study population, improving the relevance of the model for local decision-making. The internal validation of the model supports its reliability, with simulated outcomes closely aligning with observed data. In addition, the analysis incorporated a wide range of healthcare and societal costs, supporting a robust evaluation of the interventions’ value.

Nonetheless, the model has some limitations. The analyses did not account for the potential costs of implementing interventions to improve adherence, but in practice, improving or maintaining adherence may require more resources [55]. However, even with added intervention costs, the cost-effectiveness of adherence improvement would likely remain favourable, as the savings from reduced disease burden are expected to outweigh the investment [48].

Treatment effect estimates were drawn from meta-analyses, providing robust synthesis of evidence, but applying uniform relative risks across different drug classes may introduce uncertainty. Our estimates were based on the most commonly prescribed treatments in the SIDIAP database, which may overlook variation in efficacy across drug types [26, 27] and dosing regimens [30]. Similarly, applying the same relative risk across age and sex subgroups may not capture subtle differences in treatment response [57]. The model also assumes that effects of different preventive drug classes combine multiplicatively on the hazard scale, and potential pharmacological interactions between treatments are not explicitly represented, which could introduce uncertainty in the estimated overall treatment effect. We also assumed that treatment effects remain constant over time, which may not reflect real-world dynamics, particularly given the short follow-up durations of most randomised controlled trials [58]. Although adherence is a key determinant of treatment effect [4], our model may underestimate total medication impact in the observed adherence scenario, as it only applies treatment effects to patients with a PDC ≥ 80%. This may underestimate the overall effectiveness of the treatments, as patients with lower PDCs may still derive some benefit from their medication, albeit less than those with higher adherence levels [25].

As treatment effects were derived from randomised controlled trials, self-selection bias in treatment efficacy is minimised. However, adherence behaviour in real-world settings may still be influenced by patient characteristics such as baseline health status or disease severity. This behavioural self-selection could affect the generalisability of the simulated adherence scenarios to specific patient subgroups.

Moreover, the model did not account for adverse drug events, which can impact both quality of life and total healthcare costs. Incorporating adverse events in future modelling efforts would provide a more comprehensive assessment of long-term treatment value [59]. Finally, the use of routine electronic health records entails inherent limitations, such as variability in coding practices and potential misclassification of diagnoses. For example, some ICD-10 codes may overlap across disease categories, which can complicate the accurate classification of conditions and events in RWD.

## Conclusions

The findings of this study underscore the critical role of medication adherence in improving health outcomes for treatment of patients with CVD and diabetes. By leveraging RWD, the microsimulation model applied in this study provides a valuable tool for policymakers, healthcare providers and researchers to understand the long-term benefits and economic implications of adherence interventions. Future research should aim to address the identified limitations of this study and explore the long-term implications of adherence in diverse patient populations.

## Supplementary Information

Below is the link to the electronic supplementary material.

Supplementary file1 (DOCX 8139 KB)

## Declarations

### Data Availability Statement

The research team is not the data owner, as they only reused data from public health institutions. The data was used under license for the current study and are not publicly available. ESCA and SIDIAP data may be obtained upon request. The model programming code is available on an online repository [23] ensuring transparency and reproducibility of the computational methods employed in this research.
