# Supplementary material for: Lifetime Effects of Adherence to Cardiovascular and Diabetes Medications in Spain: A Modelling Study in a Population Cohort of 152,117 Patients
Source: Pharmacoeconomics. 2026 Mar 17;44(7):851–63. doi: 10.1007/s40273-026-01597-1 (PMC13290886; doi:10.1007/s40273-026-01597-1)
Supplement: Supplementary file 1 — Supplementary file1 (DOCX 8139 KB) [file 40273_2026_1597_MOESM1_ESM.docx]

**Lifetime effects of adherence to cardiovascular and diabetes medications in Spain: a modelling study in a population cohort of 152,117 patients**

***SUPPLEMENTARY MATERIAL***

[**Supplementary Methods A:** Quintiles of Deprivation Index (IP2011) 4](#_Toc219374146)

[**Supplementary Methods B.** The modelling procedure 5](#_Toc219374147)

[***Estimation of model risk equations*** 5](#_Toc219374148)

[***Estimation of transition probabilities*** 6](#_Toc219374149)

[***Integrating treatment effects in the microsimulation model*** 7](#_Toc219374150)

[**Supplementary Methods C:** Health-related quality of life 9](#_Toc219374151)

[***Table SMC1.*** *Sample characteristics of the Catalan Health Survey dataset.* 9](#_Toc219374152)

[***Table SMC2.*** *The health-related quality of life. Results of a linear regression model derived from the Catalan Health Survey from 2019 to 2022.* 10](#_Toc219374153)

[**Supplementary Methods D.** Data preparation**:** Handling of missing values 12](#_Toc219374154)

[***Table SMD1.*** *Individuals with missing data* 12](#_Toc219374155)

[**Supplementary Methods E.** Cost calculations 14](#_Toc219374156)

[***Healthcare perspective*** 14](#_Toc219374157)

[***Societal perspective*** 15](#_Toc219374158)

[***Table SME1.*** *Annual costs. Inflated to August 2024 costs in Spain.* 17](#_Toc219374159)

[**Supplementary Table ST1.** Pharmacotherapeutic groups available in the SIDIAP database which were (A) included and (B) excluded from the study, based on the Anatomical Therapeutic Chemical (ATC) Classification system. [21] 20](#_Toc219374160)

[**Supplementary Table ST2.** Diagnostics used from the SIDIAP database to classify prior diseases, utilising the International Classification of Diseases, 10th Revision (ICD-10). [22] 22](#_Toc219374161)

[**Supplementary Table ST3.** Coefficients (95% confidence interval) and hazard ratios for the risk equations estimated from the study cohort. 25](#_Toc219374162)

[**Supplementary table ST4.** TEN-SPIDERS tool reporting for proportion of days covered (PDC) calculation. 28](#_Toc219374163)

[**Supplementary Table ST5.** Relative treatment effects on cardiovascular endpoints and all-cause death based on the literature. 30](#_Toc219374164)

[**Supplementary Table ST6.** Model parameters used in the probabilistic sensitivity analyses and the assumed distributions 31](#_Toc219374165)

[**Supplementary Table ST7.** Patients with concomitant medication prescribed during the 8-year follow-up period. 36](#_Toc219374166)

[**Supplementary Table ST8.** Threshold analysis estimating the maximum cost per patient (€) of adherence-enhancing interventions that would remain cost-effective at a willingness-to-pay threshold of €25,000 per QALY, under healthcare and total cost perspectives at 3% discount rate. 37](#_Toc219374167)

[**Supplementary Table ST9.** Predicted outcomes for different discount rates for the overall sample. 38](#_Toc219374168)

[**Supplementary Figure SF1.** The proportion of patients with optimal adherence observed in the study cohort for each medication subgroup of the index prescription is stratified by sex and age group. 39](#_Toc219374169)

[**Supplementary Figure SF2.** Annual cumulative hazard over 8 years of follow-up for cardiovascular endpoints: observed data (Nelson–Aalen estimator) versus predictions from the CVD Markov model. 42](#_Toc219374170)

[**Supplementary Figure SF3.** Mean predicted outcomes and costs over a lifetime, discounted at 3% across three scenarios of medication adherence by CVD prevention, sex and age, for antihypertensive, oral glucose-lowering, lipid-lowering, and antiplatelet medication. 47](#_Toc219374171)

[**Supplementary Figure SF4.** Incremental cost per life year gained and incremental cost per QALY gained, discounted at 3% across three scenarios of medication adherence by CVD prevention, sex and age, for the overall cohort and by treatment group (antihypertensive, oral glucose-lowering, lipid-lowering, and antiplatelet medication). 52](#_Toc219374172)

[**Supplementary Figure SF5.** Cost-effectiveness planes showing results from the probabilistic sensitivity analysis (500 bootstrap replications) and deterministic analysis, with healthcare and societal costs plotted against QALYs and life-years. 56](#_Toc219374173)

[**Supplementary Figure SF6.** Cost-effectiveness acceptability curves for a willingness to pay threshold of 25.000€/QALY from the A) healthcare and B) societal perspectives. 58](#_Toc219374174)

[**Supplementary Figure SF7.** Convergence of incremental cost per LY and QALY gained estimates across probabilistic sensitivity analysis simulations. 61](#_Toc219374175)

[**CHEERS 2022 Checklist**. Adapted from Husereau et al. [24] 65](#_Toc219374176)

# **Supplementary Methods A:** Quintiles of Deprivation Index (IP2011)

The 2011 Deprivation Index of the Spanish Society of Epidemiology was published by Duque et al. [1] It is a measure that assesses the level of socio-economic deprivation in various geographic areas of Spain using the Population and Housing Census data from 2011. This index is based on socio-economic indicators such as educational level, average income, unemployment rate, and housing quality, among others. The study uses quintiles to classify geographical areas according to their level of deprivation, allowing for a relative comparison of deprivation levels across different geographical areas.

# **Supplementary Methods B.** The modelling procedure

## ***Estimation of model risk equations***

We developed risk equations for four model endpoints—stroke, coronary heart disease (CHD), heart failure (HF), and all-cause death—utilising data from the SIDIAP database. Following the specified inclusion and exclusion criteria outlined in the methods section, we ultimately included 152,117 individuals.

For stroke, CHD, and HF, incident events were identified from primary care and hospital records using the ICD-10 codes listed in Supplementary Table ST2. The event date was defined as the first qualifying diagnosis recorded after the index prescription date. Participants were followed from the index date until the earliest of endpoint occurrence, death, transfer out of the healthcare area, or administrative end of follow-up (31 December 2021). All-cause mortality was obtained from SIDIAP’s electronic health records, and deaths could occur with or without a preceding cardiovascular event.

Predictor covariates included in the risk equations were age, sex, cardiovascular risk factors (diabetes, dyslipidaemia, hypertension, smoking history, or atherosclerosis), baseline CVD history (to capture prior disease burden), and socioeconomic deprivation. Medication adherence for each pharmacotherapeutic group was also included to account for treatment exposure in the real-world population.

The mortality equation captured separately the short-term and long-term risks of death following cardiovascular events. Separate time-updated indicators for stroke, CHD, and HF were included to distinguish between deaths occurring in the same year as the event and in subsequent years, allowing the model to capture the acute mortality risk immediately after an event as well as the sustained excess risk thereafter.

We applied parametric proportional hazard models using three different distributions (exponential, Weibull, and Gompertz) to each endpoint. The best-fitting model was selected based on Akaike Information Criterion (AIC) and Bayesian Information Criterion (BIC) values. Ultimately, the Gompertz distribution was selected for modelling death, while the Weibull distribution was used for cardiovascular events.

The microsimulation model was coded in Python (version 3.13.9) using Visual Studio Code (VSC). Employing the parametric risk equations derived from the study cohort, the microsimulation model randomly simulates endpoints for each individual based on their baseline characteristics until reaching 110 years of age or death. Each annual cycle could include non-fatal or fatal events and all-cause death. Individuals experiencing an event could die within the same cycle (same year fatal event) or in subsequent cycles (post-event excess mortality), according to the estimated transition probabilities.

To evaluate the model's internal performance, we plotted the predicted cumulative risks of each event against observed risks, concluding that the incidence of death and cardiovascular events (stroke, CHD and HF) during the first 8 cycles is similar to that observed in the 8-year follow-up of the SIDIAP database

## ***Estimation of transition probabilities***

In our model, transition probabilities (*tp*) represent the likelihood of events occurring within each cycle. We calculated these probabilities using the formula$tp\left( u \right)=1-exp[H\left( t-u \right)-H(t)]$, where $u$ denotes the length of the cycle (1 year in our study), and *H(t-u)* and *H(t)* are the cumulative hazards at time *t-u* and *t*, respectively. This formula quantifies the probability of events occurring within a specific time period by comparing cumulative hazards between the start and end of the cycle. Moreover, age and prior event experiences significantly influence future event risks within the model. To account for these factors, the risk equations incorporate annually updated covariates for events. Specifically, the effect of age and previous events is integrated into the cumulative hazards using an exponential function including the coefficients that represent the impacts of new events and advancing age, which are updated in each cycle. This comprehensive approach ensures that age and prior event experiences are appropriately considered in predicting future event risks within our model.

## ***Integrating treatment effects in the microsimulation model***

Treatment effects were incorporated into the model based on medication adherence. The relative risk (RR) of major cardiovascular events—such as stroke, CHD, HF, or all-cause death—after the use of antihypertensive, lipid-lowering, oral glucose-lowering, or antiplatelet medication was derived from published meta-analyses randomised controlled trials comparing active treatment with placebo.[2–7]

Since the survival equations used to estimate transition probabilities were derived from a partially treated real-world population, the baseline hazards implicitly reflect some degree of treatment effect. To ensure that treatment effects were not double counted in the simulation, we first reconstructed a counterfactual untreated baseline before re-applying treatment effects.

For each individual and cycle (*t*), we calculated an untreated linear predictor *(*${LP}_{untreated}$) by removing the treatment effect for the treated participants: ${LP}_{untreated,t}={LP}_{observed,t}-({ADH}_{t} x\ln\left( RR \right))$, where ${LP}_{observed}$ represents the estimated linear predictor from the fitted survival equation, ${ADH}_{t}$is the individual’s annual adherence (being 0 or 1 if PDC ≥ 80%), and *RR* is the literature-based relative risk for the corresponding pharmacotherapeutic group (as detailed in Supplementary Table ST4).

From this untreated baseline, we then re-applied the literature-based treatment effects to obtain the linear predictors for each adherence scenario: ${LP}_{scenario,t}={LP}_{untreated,t}+({ADH}_{t} x\ln\left( RR \right))$, where scenarios represented “observed adherence”, as observed in the dataset, or “full adherence” (ADH=1 throughout the model). These scenario-specific linear predictors were then used to calculate annual transition probabilities using the parametric survival functions (as described in the previous section). This procedure ensures that the non-adherence scenario reflects the expected higher event incidence, while the observed- and full-adherence scenarios appropriately incorporate treatment effects without double-counting.

This approach provides a coherent framework for comparing the long-term outcomes of different adherence scenarios.

# **Supplementary Methods C:** Health-related quality of life

Instead of using the pre-existing quality of life (QoL) values from the literature, we derived cohort-specific QoL values using data from the Catalan Health Survey (Enquesta de Salut de Catalunya – ESCA). This survey is designed to represent the Catalan population, which is similar to the population attending Catalan Primary Care in terms of age, gender, socioeconomic status, and health conditions. Table SMC1 show the characteristics of the Catalan Health Survey population.

## ***Table SMC1.*** *Sample characteristics of the Catalan Health Survey dataset.*

|  | | | **2019-2022 Catalan Health Survey data** (N=12,134) |
| --- | --- | --- | --- |
| *Age ^a^ – Mean (SD)* | | | 53,97 (20.12) |
| *Age ^a^ (%)* | | |  |
|  | | *≤ 46 years old* | 4,654 (38.36) |
|  | | *47-55 years old* | 1,506 (12.41) |
|  | | *56-65 years old* | 2,432 (20.04) |
|  | | *66-76 years old* | 1,562 (12.87) |
|  | | *≥ 77 years old* | 1,980 (16.32) |
| *Sex (%)* | | |  |
|  | *Female* | | 6,205 (51.14) |
|  | *Male* | | 5,929 (48.86) |
| *Cardiovascular risk factors ^a^ (%)* | | |  |
|  | | *Diabetes* | 1,250 (10.30) |
|  | | *Dyslipidaemia* | 2,915 (24.02) |
|  | | *Hypertension* | 3,654 (30.11) |
| *History of cardiovascular events ^a^ (%)* | | |  |
|  | | Stroke ever | 194 (1.60) |
|  | | Stroke previous 12 months | 113 (0.93) |
|  | | CHD ever | 520 (4.29) |
|  | | CHD previous 12 months | 622 (5.13) |
| *Socioeconomic status ^a,b^ (%)* | | |  |
|  | High ^c^ | | 2,836 (23.37) |
|  | Medium ^d^ | | 2,214 (18.25) |
|  | Low ^e^ | | 6,782 (55.89) |
|  | Never worked | | 302 (2.49) |
| *Smoking history ^a,b^ (%)* | | |  |
|  | | *Non-smoker* | 6,821 (56.21) |
|  | | *Smoker* | 2,612 (21.53) |
|  | | *Ex-smoker* | 2,70 (22.26) |
| ^a^ Information at the time of the index prescription.  ^b^ Missing data imputed using multiple imputation by chained equations (MICE).  ^c^ Managers, careers that require a college degree, athletes and artists.  ^d^ Intermediate occupations (administrative and support workers) and self-employed workers  ^e^ Manual workers (Supervisors and workers in qualified technical occupations, qualified workers in the primary sector, semi-skilled workers and unskilled workers) | | | |

We used the pooled data from the 2019-2022 Catalan Health Survey to develop a linear regression model predicting individuals’ QoL using their characteristics, disease, and cardiovascular event history (Table SMC2).

## ***Table SMC2.*** *The health-related quality of life. Results of a linear regression model derived from the Catalan Health Survey from 2019 to 2022.*

| MODEL ^a^ | | Model on 2019-2022 Catalan Health Survey data  (N=12,134) | | | | | |
| --- | --- | --- | --- | --- | --- | --- | --- |
|  | | Coefficient | (95%CI) | | | | |
| Intercept ^b^ | | 0.914 | ( | 0.899 | - | 0.929 | ) |
| Age (centred at 55) | | -0.003 | ( | -0.003 | - | -0.003 | ) |
| Female | | -0.041 | ( | -0.050 | - | -0.033 | ) |
| Hypertension* | | -0.029 | ( | -0.038 | - | -0.020 | ) |
| Dyslipidaemia* | | -0.004 | ( | -0.013 | - | -0.005 | ) |
| Diabetes* | | -0.055 | ( | -0.067 | - | -0.042 | ) |
| CVD history | |  | | | | | |
|  | Past stroke ^c^ | -0.153 | ( | -0.182 | - | -0.124 | ) |
|  | Recent stroke ^d^ | -0.138 | ( | -0.176 | - | -0.101 | ) |
|  | Past CHD ^c^ | -0.069 | ( | -0.087 | - | -0.050 | ) |
|  | Recent CHD ^d^ | -0.092 | ( | -0.109 | - | -0.076 | ) |
| Smoking history* | |  |  |  |  |  |  |
|  | Non-smoker | Ref. |  |  |  |  |  |
|  | Smoker | 0.003 | ( | -0.028 | - | 0.033 | ) |
|  | Ex-smoker | 0.034 | ( | 0.011 | - | 0.056 | ) |
| Socioeconomic status* | |  | | | | | |
|  | High ^e^ | 0.035 | ( | 0.026 | - | 0.044 | ) |
|  | Medium ^f^ | 0.018 | ( | 0.009 | - | 0.028 | ) |
|  | Low ^g^ | Ref. |  |  |  |  |  |
|  | Never worked | -0.056 | ( | -0.079 | - | -0.032 | ) |
| CVD: Cardiovascular disease; CHD: Coronary Heart Disease  ^a^ Model constructed with a population >18 years old  ^b^ The intercept corresponds to the QoL (i.e. EQ-5D utility) of 55-year-old male from Catalonia (Spain), without history of CVD or cardiovascular events, non-smoker and with low socioeconomic status (manual worker).  ^c^ Event occurring more than a year ago  ^d^ Event occurring in the last year  ^e^ Managers, careers that require a college degree, athletes and artists.  ^f^ Intermediate occupations (administrative and support workers) and self-employed workers  ^g^ Manual workers (Supervisors and workers in qualified technical occupations, qualified workers in the primary sector, semi-skilled workers and unskilled workers)  *Variables imputed using multiple imputation by chained equations. | | | | | | | |

This model was used to predict QoL utilities for each annual cycle in our study.

Since the Catalan Health Survey dataset did not include heart failure incidence, we used literature data to estimate QoL for these patients and adjusted this value to match our cohort's demographics and clinical characteristics. Starting with a mean QoL value of 0.64 from the literature,[8] we subtracted the intercept of our regression model and adjusted for age differences (72.5 vs. 55 years old) and comorbidities such as hypertension, dyslipidaemia, diabetes, stroke, and coronary heart disease using our cohort's incidence rates and the regression model coefficients. Overall, the estimated QoL reduction for patients with heart failure in Spain was 0.184.

# **Supplementary Methods D.** Data preparation**:** Handling of missing values

The pattern of missingness was assumed to be missing at random. We applied multiple imputations by chained equations (MICE) with predictive mean matching and five nearest neighbours, with a random seed in 1993, to ensure reproducibility and address missing data.

We carefully considered the missing data rates for each dataset and the balance between computational feasibility and robustness in determining the number of imputed databases. Table SMD1 shows the proportion of missing data in the used databases.

## ***Table SMD1.*** *Individuals with missing data*

1. *At baseline in the SIDIAP database (N = 152,117)*

|  | **Nº individuals with missing values** | **Individuals with missing values (%)** |
| --- | --- | --- |
| *Socioeconomic deprivation* | 18,310 | 12.04 |
| *Smoking status* | 24,305 | 15.98 |

1. *At the Catalan Health Survey 2019-2022 (N=12,134)*

|  | **Nº individuals with missing values** | **Individuals with missing values (%)** |
| --- | --- | --- |
| *Hypertension* | 42 | 0.35 |
| *Dyslipidaemia* | 27 | 0.22 |
| *Diabetes* | 15 | 0.12 |
| *Smoking status* | 674 | 5.55 |
| *Socioeconomic status* | 173 | 1.43 |

The study cohort, extracted from the SIDIAP dataset, exhibited a moderate range of missing data rates, with values between 12% and 16% across variables. In contrast, the Catalan Health Survey dataset, used to calculate health-related quality of life, displayed lower levels of missing data, with rates up to 6% across variables. Due to the substantial size of the dataset and the computational limitations, we generated 10 imputed datasets. While this number is generally deemed sufficient for reliable estimates, higher precision could potentially be achieved with more imputations. However, practical constraints led us to settle on 10 imputed datasets, striking a balance between robustness and computational efficiency.

Our consistent approach to employing 10 imputed databases across both datasets aligns with established guidelines, offering a dependable foundation for subsequent analyses.

The dataset was reformatted into a wide format to facilitate the imputation process. In the imputation of the SIDIAP dataset, variables of socioeconomic deprivation and smoking status were registered for imputation. Baseline characteristics, including age, sex and follow-up time, treatment prescription, medication adherence during the first year after the index prescription, cardiovascular risk factors at baseline (diabetes, hypertension, hypercholesterolemia and atherosclerosis), cardiovascular events before the index prescription (stroke, coronary heart disease and heart failure) and incidence of cardiovascular events during follow-up were registered as regular variables, serving as predictors in the imputation process.

In the Catalan Health Survey dataset, the variables registered for imputation were hypertension, dyslipidaemia, diabetes, smoking and socioeconomic status. The variables registered as regular were as follows: demographic (age and sex), health status (diagnoses of diseases or medical conditions; body mass index), use of healthcare resources during the previous 12 months, medication prescribed in the previous 15 days, lifestyle (physical activity, diet, alcohol consumption), family context and sociodemographic data.

On one hand, the imputed datasets were combined following Rubin's rules to fit the regression models. Rubin's rules are used to combine the estimates from each imputed dataset, accounting for the variability between imputations to produce a single set of regression coefficients. On the other hand, for the microsimulation process, missing values of smoking status or socioeconomic deprivation were filled in by using the mode across all imputed datasets.

# **Supplementary Methods E.** Cost calculations

## ***Healthcare perspective***

The costs from the healthcare perspective included direct costs from annual treatment expenses and the yearly costs of cardiovascular disease.

The calculation of annual treatment expenses in the study includes the costs of medical visits and tests and the costs of medications.

The visits and tests were determined based on the recommendations from the clinical practice guidelines provided by the Catalan Institute of Health (ICS). Each guideline specifies the frequency and type of medical consultations and diagnostic tests necessary for proper patient management of hypertension,[9] diabetes[10] and dyslipidaemia and cardiovascular risk.[11] To calculate the annual costs of medical visits, we multiplied the number of services recommended for each disorder by their unit cost. If a patient had more than one medical condition, the recommended number of visits was based on the condition with the highest visit requirement, rather than summing the visits for each condition. Unit costs were based on the tariffs published by the Catalan Bulletin in 2023. [12]

Calculating the annual medication costs in the study involved several steps to ensure accuracy and representativeness. Initially, the three most commonly prescribed treatments for each medication group were identified from the SIDIAP dataset (listed in Table SME1). The cost per dose for each medication was then determined using the official prices of generic versions in Spain in 2024.[13] Typically, the dosage of those treatments is one pill per day, except for Metformin and its combination with Sitagliptin, which is two pills per day. These daily costs were scaled up to an annual figure considering 365 days. To accurately reflect real-world usage patterns, the annual costs were weighted based on the proportion of patients using each treatment in the SIDIAP dataset. This means that medications more frequently prescribed in the cohort had a greater influence on the overall cost calculation.

Costs of cardiovascular events were extracted from the published literature. Yearly costs associated with stroke and coronary heart disease (CHD) were divided into those incurred during the event year and those incurred in subsequent years. For the event year, we accounted for index admission cost (direct hospital cost for the acute period of the event) [14,15] and added a half-cycle correction. We assumed that the event would occur mid-year, so we added half of the subsequent annual costs to the event year. Full subsequent annual costs were applied for each following year, ensuring a more accurate estimation of the total costs over time. Annual direct medical costs for years following the event included primary care visits, specialist consultations, hospitalisations, and emergency visits. [15,16] This cost structure was applied in each cycle after the event to ensure a comprehensive view of the long-term economic impact.

On the other hand, the analysis assumed a consistent cost structure for heart failure throughout all cycles, which included outpatient, inpatient, and medication costs. [17]

## ***Societal perspective***

In addressing the societal perspective within our study, we incorporated several key elements to comprehensively assess the economic impact of cardiovascular events.

First, we included productivity losses due to morbidity related to these events, based on published data. [15–17] For stroke and CHD, we also accounted for the financial implications of informal caregiving. [15,16] Moreover, we estimated the annual cost of lost productivity due to premature mortality from cardiovascular disease by using Spain's activity rate[18] and the mean monthly salary in 2022.[19] This allowed us to calculate the economic burden associated with lost productivity before reaching Spain's retirement age of 66.[20] These costs were applied to all patients who entered the absorbing state of death in the model before the age of 66.

Table SME1 has detailed information on all costs used in the model. All costs were adjusted according to the 2023 Spanish Consumer Price Index if published before 2023.

## ***Table SME1.*** *Annual costs. Inflated to August 2024 costs in Spain.*

1. *Medication costs*

| *Cost item* | | |  |  |  |  | *Reference year of origin* | *Ref.* |
| --- | --- | --- | --- | --- | --- | --- | --- | --- |
| ***Healthcare resource use*** | | |  |  |  | ***Unit cost (€)*** |  |  |
| General practitioner (GP) visit | | |  |  |  | 65 | *2024* | [12] |
| Nurse visit | | |  |  |  | 45 | *2024* |  |
| Blood test | | |  |  |  | 85 | *2024* |  |
| HbA1c in blood test | | |  |  |  | 3.57 | *2024* |  |
| ***Active pharmaceutical ingredient*** | | | ***Tab/box*** | ***Drug price ^a^ (€)*** | ***Prevalence of use ^b^ (%)*** | ***Annual cost ^c^ (€)*** |  |  |
| **Antihypertensive treatment ^d^** | | |  |  |  | **265.69** |  |  |
|  | *Enalapril 20mg* | | 28 | *1.61* | *60* | *12.54* | *2024* | [13] |
|  | *Hydrochlorothiazide 25mg* | | 20 | *2.34* | *23* | *9.62* | *2024* |  |
|  | *Bisoprolol 5mg* | | 60 | *3.28* | *18* | *3.54* | *2024* |  |
|  | *Weighted medication cost* | |  |  |  | *25.69* |  |  |
| **Oral glucose-lowering treatment ^e^** | | |  |  |  | **384.79** |  |  |
|  | *Metformin 850mg* | | 50 | *1.94* | *87* | *24.74* | *2024* | [13] |
|  | *Gliclazide 30mg* | | 60 | *5.59* | *9* | *3.10* | *2024* |  |
|  | *Sitagliptin/metformin 50/1000mg* | | 56 | *27.32* | *4* | *12.67* | *2024* |  |
|  | *Weighted medication cost* | |  |  |  | *40.51* |  |  |
| **Lipid-lowering treatment ^d^** | | |  |  |  | **276.88** |  |  |
|  | *Simvastatin 20mg* | | 28 | *1.58* | *74* | *15.23* | *2024* | [13] |
|  | *Atorvastatin 20mg* | | 28 | *7.20* | *18* | *16.77* | *2024* |  |
|  | *Gemfibrozil 600mg* | | 60 | *9.80* | *8* | *4.87* | *2024* |  |
|  | *Weighted medication cost* | |  |  |  | *36.88* |  |  |
| **Antiplatelet treatment ^d^** | | |  |  |  | **279.92** |  |  |
|  | *Aspirin 100mg* | | 30 | *1.45* | *88* | *15.52* | *2024* | [13] |
|  | *Clopidogrel 75mg* | | 28 | *16.81* | *11* | *23.52* | *2024* |  |
|  | *Triflusal 300mg* | | 30 | *5.53* | *1* | *0.88* | *2024* |  |
|  | *Weighted medication cost* | |  |  |  | *39.92* |  |  |
| HbA1c: Glycated haemoglobin; mg: milligrams; tab: tablet | | | | | | | | |
| ^a^ | | Price in Spain for a generic box of the described treatment, as of September 2023 | | | | | | |
| ^b^ | | Prevalence based on the relative proportion of use of the 3 most used active principles observed in the cohort and scaled up to 100% of use. | | | | | | |
| ^c^ | | Assuming a dose of 1 tablet per day, except for metformin and Sitagliptin/Metformin the dose is assumed to be 2 tablets/day. Total cost is obtained by weighting the cost of each active principle by the relative prevalence of use for a whole year of treatment. | | | | | | |
| ^d^ | | The cost of treatment includes one follow-up visit with a GP, two with the nurse and one blood test annually [9,11] | | | | | | |
| ^e^ | | The cost of oral glucose-lowering treatments includes one follow-up visit with a GP, four with the nurse and one blood test with HbA1c annually [10] | | | | | | |

| *Cardiovascular event* | | ***Annual cost (€)*** *^f^*  *(Year of event)* | ***Annual healthcare cost (€)*** *^g^*  *(Follow-up)* | ***Annual cost of work morbidity-induced productivity loss (€)*** *^h^ (Follow-up)* | | ***Annual cost of informal care (€)*** *^i^ (Follow-up)* | *Reference year of origin* | *Ref.* |
| --- | --- | --- | --- | --- | --- | --- | --- | --- |
| Stroke | | 13,535.00 | 1,638.00 | | 2,388.00 | 349.00 | 2023 | [15] |
| Coronary Heart Disease | | 8,344.74^j^ | 784.14 | | 196.45 | 237.54 | 2003 | [16] |
| Heart failure | | 3,020.58^k^ | 3,020.58^k^ | | 236.23 | 0 | 2016 | [17] |
| ^f^ | The cost of the event includes the mean hospitalisation cost, and given that the event occurs half-cycle, half-annual healthcare cost is added. | | | | | | | |
| ^g^ | Direct healthcare costs during the follow-up year include primary care visits, visits to specialists, hospitalisations, emergency room visits and medication costs. | | | | | | | |
| ^h^ | Obtained after multiplying the number of days of absence from work due to sickness by the mean daily wage in Spain of a working person | | | | | | | |
| ^i^ | Lost paid and unpaid hours of informal caregiver | | | | | | | |
| ^j^ | The reference year for admission costs of ischemic heart disease is 2019. [14] | | | | | | | |
| ^k^ | Healthcare costs of heart failure do not include emergency visits. | | | | | | | |

1. *Cardiovascular event costs*
2. *Premature mortality costs*

| *Cost item* | | ***Mean annual wage in Spain (€)*** | ***Activity rate in Spain ^l^ (%)*** | ***Annual cost (€)*** | *Reference year of origin* | *Ref.* |
| --- | --- | --- | --- | --- | --- | --- |
| **Lost productivity due to premature mortality** | | 30,378.09 | 58.99 | 17,920.03 | 2022 | [19] |
| ^l^ | Labour Force Survey data: activity rate for the fourth trimester of 2023. [18] | | | | | |

# **Supplementary Table ST1.** Pharmacotherapeutic groups available in the SIDIAP database which were (A) included and (B) excluded from the study, based on the Anatomical Therapeutic Chemical (ATC) Classification system. [21]

1. *Included*

|  | *Therapeutic subgroup (2^nd^ ATC level)* | *Pharmacological subgroup*  *(3^rd^ ATC level)* |
| --- | --- | --- |
| *Oral glucose-lowering medications* | **A10 - Drugs used in diabetes** | A10B - Blood glucose lowering drugs, excluding insulins |
| *Antiplatelet medications* | **B01 - Antithrombotic agents** | B01AC - Platelet aggregation inhibitors excluding Heparin |
| *Antihypertensive medications* | **C02 - Antihypertensives** | C02C - Antiadrenergic agents, peripherally acting |
|  | **C03 - Diuretics** | C03A - Low-ceiling diuretics, thiazides |
|  |  | C03B - Low-ceiling diuretics, excl. Thiazides |
|  |  | C03C - High-ceiling diuretics |
|  |  | C03D - Potassium-sparing agents |
|  |  | C03E - Diuretics and potassium-sparing agents in combination |
|  | **C07 - Beta blocking agents** | C07A - Beta blocking agents |
|  |  | C07B - Beta blocking agents and thiazides |
|  |  | C07C - Beta blocking agents and other diuretics |
|  | **C08 - Calcium channel blockers** | C08C - Selective calcium channel blockers with mainly vascular effects |
|  | **C09 - Agents acting on the renin-angiotensin system** | C09A - ACE inhibitors, plain |
|  |  | C09B - ACE inhibitors, combinations |
|  |  | C09C - Angiotensin II receptor blockers (ARBS), plain |
|  |  | C09D - ARBS, combinations |
|  |  | C09X - Other agents acting on the renin-angiotensin system |
| *Lipid-lowering medications* | **C10 - Lipid modifying agents** | C10A - Lipid modifying agents, plain |

1. *Excluded*

|  | *Therapeutic subgroup (2^nd^ ATC level)* | *Pharmacological subgroup*  *(3^rd^ ATC level)* | *Reason for exclusion* |
| --- | --- | --- | --- |
| *Oral glucose-lowering medications* | **A10 - Drugs used in diabetes** | A10A – Insulins and analogues | Incorrect dosing information in the database, hampering adherence calculation. These prescriptions accounted for  < 2% of the total. |
| *Antihypertensive medications* | **C02 - Antihypertensives** | C02A - Antiadrenergic agents, centrally acting | Mainly used in pregnant women. |
|  |  | C02D - Arteriolar smooth muscle, agents acting on |  |
|  | **C07 - Beta blocking agents** | C07D - Beta blocking agents, thiazides and other diuretics | Combinations are not used as first-line treatment.  Used when monotherapy does not work. |
|  |  | C07F - Beta blocking agents, other combinations |  |
|  | **C08 - Calcium channel blockers** | C08D - Selective calcium channel blockers with direct cardiac effects | Indicated for other conditions. |
| *Lipid-lowering medications* | **C10 - Lipid modifying agents** | C10B - lipid modifying agents, combinations | Indicated for secondary prevention.  Not included in prescribing quality standards of the Catalan Institute of Health. |

# **Supplementary Table ST2.** Diagnostics used from the SIDIAP database to classify prior diseases, utilising the International Classification of Diseases, 10th Revision (ICD-10). [22]

| *Diagnosis* | *ICD-10 code* | *Description* |
| --- | --- | --- |
| *Stroke* | **G45_9** | Transient cerebral ischemic attack, unspecified |
|  | **I60** | Non-traumatic subarachnoid haemorrhage |
|  | **I60_9** | Non-traumatic subarachnoid haemorrhage, unspecified |
|  | **I61_0** | Non-traumatic intracerebral haemorrhage |
|  | **I61_1** | Non-traumatic intracerebral haemorrhage in hemisphere, cortical |
|  | **I61_2** | Non-traumatic intracerebral haemorrhage in hemisphere, unspecified |
|  | **I61_3** | Non-traumatic intracerebral haemorrhage in brain stem |
|  | **I61_4** | Non-traumatic intracerebral haemorrhage in cerebellum |
|  | **I61_5** | Non-traumatic intracerebral haemorrhage, intraventricular |
|  | **I61_8** | Other non-traumatic intracerebral haemorrhage |
|  | **I61_9** | Non-traumatic intracerebral haemorrhage, unspecified |
|  | **I62_1** | Non-traumatic extradural haemorrhage |
|  | **I62_9** | Non-traumatic intracranial haemorrhage, unspecified |
|  | **I63** | Cerebral infarction |
|  | **I63_3** | Cerebral infarction due to thrombosis of carotid artery |
|  | **I63_8** | Other cerebral infarction |
|  | **I63_9** | Cerebral infarction, unspecified |
|  | **I65_1** | Occlusion and stenosis of basilar artery |
|  | **I65_2** | Occlusion and stenosis of carotid artery |
|  | **I65_9** | Occlusion and stenosis of unspecified pre-cerebral artery |
|  | **I66_3** | Occlusion and stenosis of cerebellar arteries |
|  | **I66_8** | Occlusion and stenosis of other cerebral arteries |
|  | **I66_9** | Occlusion and stenosis of unspecified cerebral artery |
|  | **I67_0** | Dissection of cerebral arteries, non-ruptured |
|  | **I67_9** | Cerebrovascular disease, unspecified |
| *Coronary heart disease* | **I20_0** | Unstable angina |
|  | **I20_1** | Angina pectoris with documented spasm |
|  | **I20_8** | Other forms of angina pectoris |
|  | **I20_9** | Angina pectoris, unspecified |
|  | **I21** | Acute myocardial infarction |
|  | **I21_0** | ST elevation (STEMI) myocardial infarction of anterior wall |
|  | **I21_3** | ST elevation (STEMI) myocardial infarction of unspecified site |
|  | **I21_4** | Non-ST elevation (NSTEMI) myocardial infarction |
|  | **I21_9** | Acute myocardial infarction, unspecified |
|  | **I22_0** | Subsequent ST elevation (STEMI) myocardial infarction of anterior wall |
|  | **I22_1** | Subsequent ST elevation (STEMI) myocardial infarction of inferior wall |
|  | **I22_8** | Subsequent ST elevation (STEMI) myocardial infarction of other sites |
|  | **I22_9** | Subsequent ST elevation (STEMI) myocardial infarction of unspecified site |
|  | **I23_1** | Atrial septal defect as current complication following acute myocardial infarction |
|  | **I23_3** | Rupture of cardiac wall without hemopericardium as current complication following acute myocardial infarction |
|  | **I23_4** | Rupture of chordae tendineae as current complication following acute myocardial infarction |
|  | **I23_6** | Thrombosis of atrium, auricular appendage, and ventricle as current complications following acute myocardial infarction |
|  | **I24_0** | Acute coronary thrombosis not resulting in myocardial infarction |
|  | **I24_8** | Other forms of acute ischemic heart disease |
|  | **I24_9** | Acute ischemic heart disease, unspecified |
|  | **I25_6** | Silent myocardial ischemia |
| *Heart failure* | **I50_1** | Left ventricular failure, unspecified |
|  | **I50_9** | Heart failure, unspecified |
| *Diabetes* | **E10_8** | Type 1 diabetes mellitus with unspecified complications |
|  | **E10_9** | Type 1 diabetes mellitus without complications |
|  | **E11** | Type 2 diabetes mellitus |
|  | **E11_3** | Type 2 diabetes mellitus with ophthalmic complications |
|  | **E11_8** | Type 2 diabetes mellitus with unspecified complications |
|  | **E11_9** | Type 2 diabetes mellitus without complications |
|  | **E13_8** | Other specified diabetes mellitus with unspecified complications |
|  | **E13_9** | Other specified diabetes mellitus without complications |
| *Dyslipidaemia* | **E78** | Disorders of lipoprotein metabolism and other lipidaemias |
|  | **E78_0** | Pure hypercholesterolemia |
|  | **E78_1** | Pure hyperglyceridaemia |
|  | **E78_2** | Mixed hyperlipidaemia |
|  | **E78_3** | Hyperchylomicronaemia |
|  | **E78_4** | Other hyperlipidaemia |
|  | **E78_5** | Hyperlipidaemia, unspecified |
|  | **E78_6** | Lipoprotein deficiency |
|  | **E78_9** | Disorder of lipoprotein metabolism, unspecified |
| *Hypertension* | **I10** | Essential (primary) hypertension |
|  | **I11** | Hypertensive heart disease |
|  | **I11_0** | Hypertensive heart disease with heart failure |
|  | **I11_9** | Hypertensive heart disease without heart failure |
|  | **I12_0** | Hypertensive chronic kidney disease with stage 5 chronic kidney disease or end stage renal disease |
|  | **I12_9** | Hypertensive chronic kidney disease with stage 1 through stage 4 chronic kidney disease, or unspecified chronic kidney disease |
|  | **I13_0** | Hypertensive heart and chronic kidney disease with heart failure and stage 1 through stage 4 chronic kidney disease, or unspecified chronic kidney disease |
|  | **I13_2** | Hypertensive heart and chronic kidney disease with heart failure and with stage 5 chronic kidney disease, or end stage renal disease |
|  | **I15_0** | Renovascular hypertension |
|  | **I15_1** | Hypertension secondary to other renal disorders |
|  | **I15_2** | Hypertension secondary to endocrine disorders |
|  | **I15_8** | Other secondary hypertension |
|  | **I15_9** | Secondary hypertension, unspecified |
| *Atherosclerosis* | **I67_2** | Cerebral atherosclerosis |
|  | **I70_0** | Atherosclerosis of aorta |
|  | **I70_1** | Atherosclerosis of renal artery |
|  | **I70_8** | Atherosclerosis of other arteries |
|  | **I73_9** | Peripheral vascular disease, unspecified |
|  | **I74_2** | Embolism and thrombosis of arteries of the upper extremities |
|  | **I74_3** | Embolism and thrombosis of arteries of the lower extremities |
|  | **I74_4** | Embolism and thrombosis of arteries of extremities, unspecified |
|  | **I74_8** | Embolism and thrombosis of other arteries |
|  | **I74_9** | Embolism and thrombosis of unspecified artery |
|  | **I77_1** | Stricture of artery |

# **Supplementary Table ST3.** Coefficients (95% confidence interval) and hazard ratios for the risk equations estimated from the study cohort.

| *Endpoint* | *All-cause death* | | | | | *Stroke* | | | | | | | *Coronary heart disease* | | | | | | | | | | *Heart failure* | | | | | | | | | |  |
| --- | --- | --- | --- | --- | --- | --- | --- | --- | --- | --- | --- | --- | --- | --- | --- | --- | --- | --- | --- | --- | --- | --- | --- | --- | --- | --- | --- | --- | --- | --- | --- | --- | --- |
| *Functional form* | **Gompertz** | | | | | **Weibull** | | | | | | | **Weibull** | | | | | | | | | | **Weibull** | | | | | | | | | |  |
| *Parameters* | *Hazard ratio* | *95% CI* | | | | *Hazard ratio* | | *95% CI* | | | | | *Hazard ratio* | | | *95% CI* | | | | | | | *Hazard ratio* | | | | | *95% CI* | | | | |  |
| *Sex (Female)* | 0.75 | ( | 0.72 | 0.77 | ) | | 0.74 | | ( | 0.70 | 0.78 | ) | | 0.46 | | | ( | | 0.42 | | 0.50 | | | ) | | | 0.88 | | ( | 0.81 | 0.96 | ) | |
| *Mean age* | 1.11 | ( | 1.10 | 1.11 | ) | | 1.05 | | ( | 1.05 | 1.05 | ) | | 1.02 | | | ( | | 1.02 | | 1.02 | | | ) | | | 1.10 | | ( | 1.10 | 1.11 | ) | |
| *Current smoker* | 2.16 | ( | 2.05 | 2.27 | ) | | 1.41 | | ( | 1.30 | 1.53 | ) | | 1.50 | | | ( | | 1.37 | | 1.65 | | | ) | | | 1.38 | | ( | 1.20 | 1.58 | ) | |
| *Ex-smoker* | 1.46 | ( | 1.39 | 1.52 | ) | | 1.08 | | ( | 1.00 | 1.18 | ) | | 1.12 | | | ( | | 1.00 | | 1.24 | | | ) | | | 1.15 | | ( | 1.02 | 1.30 | ) | |
|  |  |  |  |  |  | |  | |  |  |  |  | |  | | |  | |  | |  | | |  | | |  | |  |  |  |  | |
| ***Socioeconomic Deprivation ^a^*** | | | | | | | | | | | | | | | | | | | | | | | | | | | | | | | | |  |
| *Low* | 0.87 | ( | 0.83 | 0.92 | ) | | 0.83 | | ( | 0.75 | 0.91 | ) | | 0.93 | | | ( | | 0.83 | | 1.05 | | | ) | | | 0.88 | | ( | 0.76 | 1.02 | ) | |
| *Low intermediate* | 0.92 | ( | 0.88 | 0.96 | ) | | 1.01 | | ( | 0.94 | 1.09 | ) | | 0.92 | | | ( | | 0.83 | | 1.01 | | | ) | | | 1.05 | | ( | 0.94 | 1.16 | ) | |
| *Intermediate* | 1.02 | ( | 0.97 | 1.07 | ) | | 1.02 | | ( | 0.94 | 1.12 | ) | | 0.95 | | | ( | | 0.85 | | 1.05 | | | ) | | | 1.00 | | ( | 0.89 | 1.13 | ) | |
| *High intermediate* | 1.13 | ( | 1.04 | 1.23 | ) | | 1.06 | | ( | 0.92 | 1.22 | ) | | 1.07 | | | ( | | 0.89 | | 1.28 | | | ) | | | 1.07 | | ( | 0.86 | 1.33 | ) | |
|  |  |  |  |  |  | |  | |  |  |  |  | |  | | |  | |  | |  | | |  | | |  | |  |  |  |  | |
| ***Baseline disease history*** | | | | | | | | | | | | | | | | | | | | | | | | | | | | | | | | |  |
| *Diabetes* | 1.37 | ( | 1.31 | 1.44 | ) | | 1.28 | | ( | 1.18 | 1.38 | ) | | 1.28 | | | ( | | 1.15 | | 1.43 | | | ) | | | 1.50 | | ( | 1.35 | 1.67 | ) | |
| *Dyslipidaemia* | 0.84 | ( | 0.81 | 0.87 | ) | | 1.06 | | ( | 0.99 | 1.13 | ) | | 1.59 | | | ( | | 1.45 | | 1.74 | | | ) | | | 0.95 | | ( | 0.86 | 1.04 | ) | |
| *Hypertension* | 1.23 | ( | 1.18 | 1.28 | ) | | 1.30 | | ( | 1.21 | 1.39 | ) | | 1.89 | | | ( | | 1.72 | | 2.07 | | | ) | | | 2.05 | | ( | 1.83 | 2.30 | ) | |
| *Stroke* | 1.61 | ( | 1.51 | 1.71 | ) | | 2.02 | | ( | 1.81 | 2.24 | ) | | 0.57 | | | ( | | 0.46 | | 0.70 | | | ) | | | 1.29 | | ( | 1.11 | 1.50 | ) | |
| *Heart failure* | 2.32 | ( | 2.12 | 2.53 | ) | | 1.12 | | ( | 0.91 | 1.39 | ) | | 1.37 | | | ( | | 1.02 | | 1.83 | | | ) | | | 0.34 | | ( | 0.24 | 0.49 | ) | |
| *Coronary heart disease* | 1.38 | ( | 1.26 | 1.51 | ) | | 0.67 | | ( | 0.56 | 0.80 | ) | | 1.70 | | | ( | | 1.46 | | 1.98 | | | ) | | | 1.51 | | ( | 1.25 | 1.82 | ) | |
| *Atherosclerosis* | 1.64 | ( | 1.49 | 1.81 | ) | | 0.93 | | ( | 0.76 | 1.14 | ) | | 0.81 | | | ( | | 0.62 | | 1.06 | | | ) | | | 1.56 | | ( | 1.23 | 1.98 | ) | |
|  |  |  |  |  |  | |  | |  |  |  |  | |  | | |  | |  | |  | | |  | | |  | |  |  |  |  | |
| ***Cardiovascular event history within the model*** | | | | | | | | | | | | | | | | | | | | | | | | | | | | | | | | |  |
| *Stroke* |  |  |  |  |  | | N/A | | | | | | | 0.96 | | | ( | | 0.78 | | 1.18 | | | ) | | | 1.30 | | ( | 1.11 | 1.54 | ) | |
| *Stroke – year of event* | 1.80 | ( | 1.61 | 2.01 | ) | |  | |  |  |  |  | |  | | |  | |  | |  | | |  | | |  | | | | | | |
| *Stroke – history of event* | 1.72 | ( | 1.51 | 1.95 | ) | |  | |  |  |  |  | |  | | |  | |  | |  | | |  | | |  | | | | | | |
| *Heart failure (HF)* |  |  |  |  |  | | 1.32 | | ( | 1.11 | 1.57 | ) | | 1.56 | | | ( | | 1.21 | | 2.01 | | | ) | | | N/A | | | | | | |
| *HF – year of event* | 2.16 | ( | 1.93 | 2.41 | ) | |  | |  |  |  |  | |  | | | | | | | | | | |  | | | |  |  |  |  | |
| *HF – history of event* | 2.75 | ( | 2.45 | 3.09 | ) | |  | |  |  |  |  | |  | | | | | | | | | | |  | | | |  |  |  |  | |
| *Coronary heart disease (CHD)* |  |  |  |  |  | | 1.05 | | ( | 0.86 | 1.28 | ) | | N/A | | | | | | | | | | | 2.99 | | | | ( | 2.49 | 3.59 | ) | |
| *CHD – year of event* | 1.21 | ( | 0.97 | 1.50 | ) | |  | |  |  |  |  | |  | | | | | | | | | | |  | | | |  |  |  |  | |
| *CHD – history of event* | 1.35 | ( | 1.09 | 1.69 | ) | |  | |  |  |  |  | |  | | | | | | | | | | |  | | | |  |  |  |  | |
|  |  |  |  |  |  | |  | |  |  |  |  | |  | | | | | | | | | | |  | | | |  |  |  |  | |
| ***Treatment adherence*** | | | | | | | | | | | | | | | | | | | | | | | | | | | | | | | | |  |
| *Antihypertensives* | 0.83 | ( | 0.80 | 0.87 | ) | | 0.92 | | ( | 0.86 | 0.99 | ) | | 0.95 | ( | | | 0.87 | | 1.04 | | ) | | | | 1.37 | | | ( | 1.26 | 1.48 | ) | |
| *Oral glucose-lowering* | 0.88 | ( | 0.80 | 0.97 | ) | | 1.19 | | ( | 1.03 | 1.37 | ) | | 0.99 | ( | | | 0.82 | | 1.20 | | ) | | | | 1.06 | | | ( | 0.87 | 1.29 | ) | |
| *Lipid-lowering* | 0.57 | ( | 0.54 | 0.60 | ) | | 1.08 | | ( | 1.00 | 1.17 | ) | | 1.14 | ( | | | 1.03 | | 1.26 | | ) | | | | 1.05 | | | ( | 0.93 | 1.18 | ) | |
| *Antiplatelet* | 1.08 | ( | 1.03 | 1.14 | ) | | 2.13 | | ( | 1.95 | 2.33 | ) | | 3.35 | ( | | | 2.98 | | 3.76 | | ) | | | | 1.14 | | | ( | 1.01 | 1.28 | ) | |
| *Shape** | 0.02 | ( | 0.02 | 0.03 | ) | | 0.74 | | ( | 0.72 | 0.76 | ) | | 0.78 | ( | | | 0.75 | | 0.81 | | ) | | | | 0.68 | | | ( | 0.65 | 0.70 | ) | |
| *Intercept** | -5.99 | ( | -6.06 | -5.92 | ) | | -5.60 | | ( | -5.70 | -5.50 | ) | | -6.36 | ( | | | -6.49 | | -6.23 | | ) | | | | -7.51 | | | ( | -7.67 | -7.35 | ) | |
| ^a^ Deprivation Index 2011 of the Spanish Society of Epidemiology (IP2011) [1]  *Coefficient untransformed.  N/A, not applicable  *Adherence was measured annually if a patient had an active prescription, and patients were considered adherent if PDC≥80%.* *Please note that treatment adherence (antihypertensives, lipid-lowering, antiplatelet, and glucose-lowering medications) parameters were included to better estimate individual participants risks. However, they are subject to confounding and are not used to evaluate treatment effects of medications, which are instead informed from the ramdomised controlled trials-based literature.* | | | | | | | | | | | | | | | | | | | | | | | | | | | | | | | | |  |

# **Supplementary table ST4.** TEN-SPIDERS tool reporting for proportion of days covered (PDC) calculation.

Adapted from Dalli et al. [23]

| *Parameter* | *Implementation in the study* |
| --- | --- |
| **Threshold** | Adherence was calculated as an annual PDC and dichotomised to classify patients as adherent (PDC ≥ 80%). The 80% threshold was selected as it is widely used in cardiovascular medication adherence research, however, a lower threshold of PDC ≥ 60% was explored in sensitivity analyses to assess the robustness of results to the choice of adherence cut-off. In this study, the binary adherence indicator was used to determine whether treatment effects were applied in the model. |
| **Eligibility criteria for inclusion in sample** | PDC was calculated for adults (≥18 years) initiating antihypertensive, lipid-lowering, oral glucose-lowering, or antiplatelet therapy in public primary care in Catalonia between 2012 and 2013. Only new prescriptions were included (no prescription from the same ATC third-level subgroup in the previous 6 months). Prescriptions with <100 days’ intended duration were excluded. A minimum follow-up of one year in the database was required. |
| **Numerator and denominator** | **Numerator**: number of days covered by medication supply within a calendar year, derived from dispensing records.  **Denominator**: number of days in that calendar year.  When patients received more than one prescription within the same pharmacotherapeutic group in a given year, annual PDC was calculated as a weighted average of prescription-specific PDCs, with weights proportional to the duration of each prescription. |
| **Survival** | Annual PDC was calculated within observed follow-up; no additional truncation of the annual denominator beyond censoring at end of follow-up was applied. |
| **Presupply** | No explicit presupply period was defined. Medication coverage was constructed from observed dispensing dates within each calendar year |
| **In-hospital supply** | Medication supplied during hospital admissions was not captured in outpatient pharmacy dispensing records and therefore could not be explicitly accounted for in the PDC calculation. |
| **Dosing information** | Prescribed dosing information was available for most prescriptions and typically indicated one unit per day (1 pill/24 h). Accordingly, days of coverage were derived assuming one pill per day across treatments. |
| **Early refills** | Dispensing records occurring on the same date within an ATC group were merged, preventing double counting of medication coverage. |
| **Switching** | Therapeutic switching within the same pharmacotherapeutic group was handled at the class level. Medication use was organised into treatment episodes, which were merged when the gap between consecutive episodes was ≤60 days. When consecutive episodes overlapped in time (e.g., due to switching or refilling before the end of the previous supply), the start date of the subsequent episode was shifted forward so that days of coverage were not double counted. Gaps >60 days were treated as the start of a new episode. New prescriptions for treatment initiation were defined separately using a 182-day washout period (no active prescription in the same pharmacological group in the previous 6 months). For patients receiving multiple subclasses within the same pharmacotherapeutic group, adherence at group level was summarised as a duration-weighted average across subclasses. |

# **Supplementary Table ST5.** Relative treatment effects on cardiovascular endpoints and all-cause death based on the literature.

| *Treatment* | | *Relative risk* | | | | | | | | | | | |  |
| --- | --- | --- | --- | --- | --- | --- | --- | --- | --- | --- | --- | --- | --- | --- |
|  | | **Stroke** | *95% CI* | *Ref* | **Coronary Heart disease** | *95% CI* | *Ref* | **Heart failure** | *95% CI* | *Ref* | **Death** | *95% CI* | *Ref* | |
| *Antihypertensive treatment* ^a^ | | 0.72 | 0.64-0.81 | [2] | 0.80 | 0.73-0.88 | [2] | 0.82 | 0.69-0.98 | [2] | 0.88 | 0.81-0.96 | [2] | |
| *Oral glucose-lowering treatment* ^b^ | | 0.93 | 0.89-0.98 | [3] | 0.92 | 0.88-0.96 | [3] | 0.98 | 0.90-1.08 | [3] | 0.94 | 0.90-0.98 | [3] | |
| *Lipid-lowering treatment* ^c^ | | 0.85 | 0.80-0.91 | [4] | 0.73 | 0.70-0.77 | [4] | 0.92 | 0.85-0.99 | [5] | 0.90 | 0.87-0.93 | [4] | |
| *Antiplatelet treatment* ^d^ | | 0.89 | 0.82-0.97 | [6] | 0.80 | 0.73-0.88 | [6] | 0.93 | 0.76-1.13 | [7] | 0.95 | 0.88-1.02 | [6] | |
| ^a^ | Angiotensin-converting enzyme (ACE) inhibitors vs placebo | | | | | | | | | | | | | |
| ^b^ | Glucose-lowering drugs or strategies versus standard care | | | | | | | | | | | | | |
| ^c^ | Statin vs placebo | | | | | | | | | | | | | |
| ^d^ | Aspirin vs no treatment | | | | | | | | | | | | | |

# **Supplementary Table ST6.** Model parameters used in the probabilistic sensitivity analyses and the assumed distributions

| ***Variable*** | | ***Assumed distribution*** | ***Parameters*** | | | ***Value and PSA Range*** | | | | | | | ***Note*** |
| --- | --- | --- | --- | --- | --- | --- | --- | --- | --- | --- | --- | --- | --- |
| *Utilities* | |  | *SE* | *α* | *β* | *Mean* | *99% CI* | | | | |  |  |
| QoL reduction for patients with heart failure in Spain | | 1- Gamma | 0.01 | 31,244.07 | 0.04 | -0.18 | ( | -0.16 | -0.21 | | ) | Average QoL calculated for patients with heart failure in Spain (0.64 for patients with a mean age of 72.5 years) [8] | |
| *Treatment effects* | |  | *SE* | *ln(mean)* | *SE ln(mean)* |  |  |  |  |  | |  |  |
| Relative risk of stroke after using ACE inhibitors vs placebo | | Lognormal | 0.04 | -0.33 | 0.06 | 1.06 | ( | 0.60 | 0.86 | | ) |  | |
| Relative risk of CHD after using ACE inhibitors vs placebo | | Lognormal | 0.04 | -0.22 | 0.05 | 1.05 | ( | 0.69 | 0.92 | | ) |  | |
| Relative risk of heart failure after using ACE inhibitors vs placebo | | Lognormal | 0.07 | -0.20 | 0.08 | 1.09 | ( | 0.63 | 1.06 | | ) |  | |
| Relative risk of all-cause mortality after using ACE inhibitors vs placebo | | Lognormal | 0.04 | -0.13 | 0.04 | 1.04 | ( | 0.77 | 1.00 | | ) |  | |
| Relative risk of stroke after using glucose-lowering drugs or strategies vs standard care | | Lognormal | 0.02 | -0.07 | 0.02 | 1.02 | ( | 0.86 | 1.00 | | ) |  | |
| Relative risk of myocardial infraction after using glucose-lowering drugs or strategies vs standard care | | Lognormal | 0.02 | -0.08 | 0.02 | 1.02 | ( | 0.86 | 0.98 | | ) |  | |
| Relative risk of heart failure after using glucose-lowering drugs or strategies vs standard care | | Lognormal | 0.05 | -0.02 | 0.04 | 1.05 | ( | 0.85 | 1.13 | | ) |  | |
| Relative risk of all-cause mortality after using glucose-lowering drugs or strategies vs standard care | | Lognormal | 0.02 | -0.06 | 0.02 | 1.02 | ( | 0.88 | 1.00 | | ) |  | |
| Relative risk of any first stroke after using aspirin vs no treatment | | Lognormal | 0.04 | -0.12 | 0.04 | 1.04 | ( | 0.78 | 1.01 | | ) |  | |
|  | Relative risk of a major coronary event after using aspirin vs no treatment | Lognormal | 0.04 | -0.22 | 0.05 | 1.05 | ( | 0.69 | 0.92 | | ) |  | |
|  | Relative risk of heart failure after using aspirin vs no treatment | Lognormal | 0.09 | -0.08 | 0.09 | 1.10 | ( | 0.70 | 1.23 | | ) |  | |
|  | Relative risk of all-cause mortality after using aspirin vs no treatment | Lognormal | 0.04 | -0.05 | 0.04 | 1.04 | ( | 0.85 | 1.06 | | ) |  | |
|  | Relative risk of any stroke after using statin vs placebo | Lognormal | 0.03 | -0.16 | 0.03 | 1.03 | ( | 0.77 | 0.94 | | ) |  | |
|  | Relative risk of any major coronary event after using statin vs placebo | Lognormal | 0.02 | -0.31 | 0.02 | 1.02 | ( | 0.68 | 0.79 | | ) |  | |
|  | Relative risk of first composite heart failure outcome after using statin vs placebo | Lognormal | 0.04 | -0.08 | 0.04 | 1.04 | ( | 0.82 | 1.03 | | ) |  | |
|  | Relative risk of all-cause mortality after using statin vs placebo | Lognormal | 0.02 | -0.11 | 0.02 | 1.02 | ( | 0.85 | 0.95 | | ) |  | |
| *Costs (€)* | |  | *SE* | *α* | *β* |  |  |  |  | |  |  | |
|  | Mean admission costs of stroke | Gamma | 813.85 | 26,759.21 | 49.08 | 13,535.00 | ( | 1,1159.15 | 1,6190.44 | | ) |  | |
|  | Mean direct medical costs per annum after a stroke | Gamma | 119.39 | 188.25 | 0.51 | 1,638.00 | ( | 1,293.71 | 2,031.85 | | ) |  | |
|  | Mean indirect costs per annum after stroke | Gamma | 329.30 | 111.76 | 45.20 | 2,737.00 | ( | 1,845.98 | 3,884.52 | | ) | Morbidity-related productivity loss and cost of indirect care | |
|  | Mean admission costs of ischemic heart disease | Gamma | 34.64 | 1,275,455.82 | 0.01 | 8,344.74 | ( | 8,243.22 | 8,457.37 | | ) |  | |
|  | Mean direct medical costs per annum per patient with coronary heart disease | Gamma | 55.20 | 201.81 | 3.89 | 784.14 | ( | 624.57 | 965.85 | | ) |  | |
|  | Mean indirect costs per annum per patient with coronary heart disease | Gamma | 30.55 | 403.61 | 2.15 | 433.99 | ( | 345.67 | 534.56 | | ) | Morbidity-related productivity loss and cost of indirect care | |
|  | Mean direct medical costs per annum per patient with heart failure | Gamma | 37.22 | 183,069.74 | 0.52 | 3,020.58 | ( | 2,907.04 | 3,137.09 | | ) |  | |
|  | Mean indirect costs per annum per patient with heart failure | Gamma | 5.58 | 1,792.38 | 0.13 | 236.23 | ( | 219.36 | 253.85 | | ) | Morbidity-related productivity loss and cost of indirect care | |
|  | ACE Inhibitors: Angiotensin-converting enzyme inhibitors; CHD: Coronary Heart Disease; QoL: Quality of Life | | | | | | | | | | | | |

# **Supplementary Table ST7.** Patients with concomitant medication prescribed during the 8-year follow-up period.

|  | **Treatment at entry** | | | |
| --- | --- | --- | --- | --- |
| **Treatments prescribed during follow-up** | *Antihypertensive treatment*  *N=*90,485 | *Oral glucose-lowering treatment*  *N=*17,373 | *Lipid-lowering treatment*  *N=*63,207 | *Antiplatelet treatment*  *N=20,675* |
| *Antihypertensive treatment, N (%)* | N/A | 5,884 (33.87) | 17,582 (27.82) | 11,327 (54.79) |
| *Oral glucose-lowering treatment, N (%)* | 5,935 (6.56) | N/A | 5,932 (9.39) | 2,271 (10.72) |
| *Lipid-lowering treatment, N (%)* | 17,898 (19.78) | 6,068 (34.93) | N/A | 9,979 (48.27) |
| *Antiplatelet treatment, N (%)* | 11,556 (12.77) | 2,269 (13.06) | 10,015 (15.84) | N/A |

Some patients have more than one treatment prescribed at baseline

N/A, not applicable

# **Supplementary Table ST8.** Threshold analysis estimating the maximum cost per patient (€) of adherence-enhancing interventions that would remain cost-effective at a willingness-to-pay threshold of €25,000 per QALY, under healthcare and total cost perspectives at 3% discount rate.

| **Scenario** | **Costs** | **Average maximum intervention cost**  **(€/patient)** | **SD** | **Min (€/patient)** | **Max (€/patient)** |
| --- | --- | --- | --- | --- | --- |
| Observed vs. non-adherence | Total costs | 4,184 | 555 | 2,570 | 5,584 |
| Observed vs. non-adherence | Healthcare costs | 4,041 | 546 | 2,447 | 5,411 |
| Full vs. non-adherence | Total costs | 10,098 | 1,810 | 4,813 | 14,602 |
| Full vs. non-adherence | Healthcare costs | 9,711 | 1,792 | 4,502 | 14,115 |
| Full vs. Observed adherence | Total costs | 5,914 | 1,300 | 2,234 | 9,018 |
| Full vs. Observed adherence | Healthcare costs | 5,671 | 1,288 | 2,037 | 8,709 |

# **Supplementary Table ST9.** Predicted outcomes for different discount rates for the overall sample.

| *Discount* | **Non-adherence** | | **Observed adherence** | | **Full adherence** | |
| --- | --- | --- | --- | --- | --- | --- |
| **Undiscounted**, mean (95% CI) | Mean ^a^ | 95% CI ^b^ | Mean ^a^ | 95% CI ^b^ | Mean ^a^ | 95% CI ^b^ |
| *Life Years (LY)* | 25.36 | (25.32; 25.38) | 25.73 | (25.68; 25.74) | 26.53 | (26.49; 26.56) |
| *Quality Adjusted Life Years (QALY)* | 19.14 | (18.93; 18.99) | 19.58 | (19.38; 19.43) | 20.46 | (20.26; 20.32) |
| *Lifetime Healthcare costs (€)* | 8,379 | (8,376; 8,431) | 13,014 | (13,015; 13,067) | 24,488 | (24,498; 24,550) |
| *Lifetime Healthcare + Social costs (€)* | 15,920 | (15,854; 15,984) | 20,354 | (20,296 20,423) | 31,457 | (31,416; 31,538) |
| **5% Discount**, mean (95% CI) |  |  |  |  |  |  |
| *Life Years (LY)* | 14.20 | (14.17; 14.18) | 14.33 | (14.30; 14.32) | 14.59 | (14.56; 14.57) |
| *Quality Adjusted Life Years (QALY)* | 11.17 | (11.13; 11.14) | 11.36 | (11.32; 11.34) | 11.67 | (11.64; 11.65) |
| *Lifetime Healthcare costs (€)* | 4,028 | (4,021; 4,045) | 5,806 | (5,802; 5,824) | 9,860 | (9,860; 9,882) |
| *Lifetime Healthcare + Social costs (€)* | 7,449 | (7,414; 7,470) | 9,107 | (9,077; 9,131) | 12,965 | (12,942; 12,994) |
| ^a^ Mean of the deterministic analysis  ^b^ 95% CI of 500 bootstraps from sensitivity analysis | | | | | | |

# **Supplementary Figure SF1.** The proportion of patients with optimal adherence observed in the study cohort for each medication subgroup of the index prescription is stratified by sex and age group.

*Censored data is not represented in the figures.*


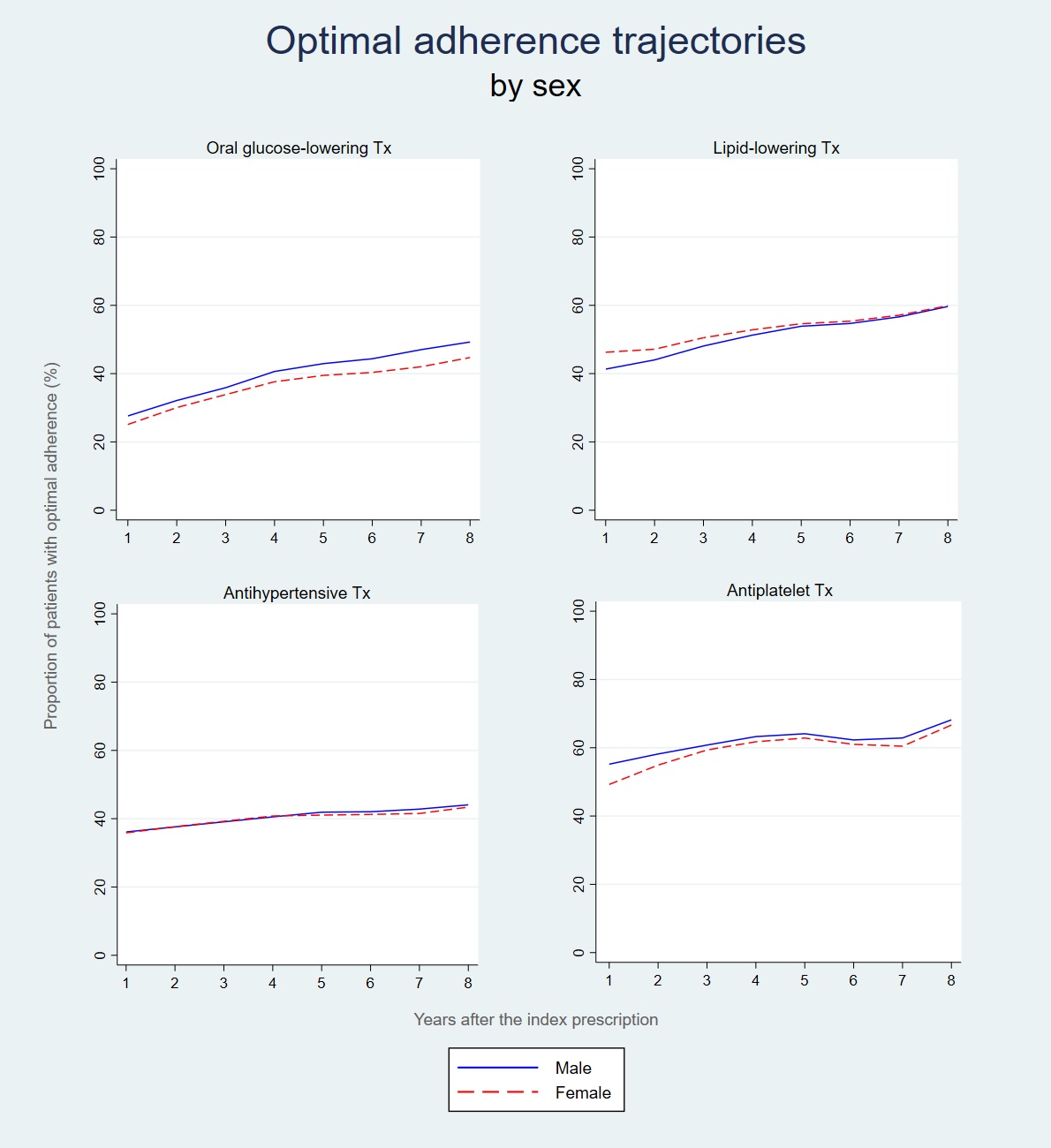


Tx: treatment

Optimal adherence is defined as an annual proportion of days covered ≥ 80%.


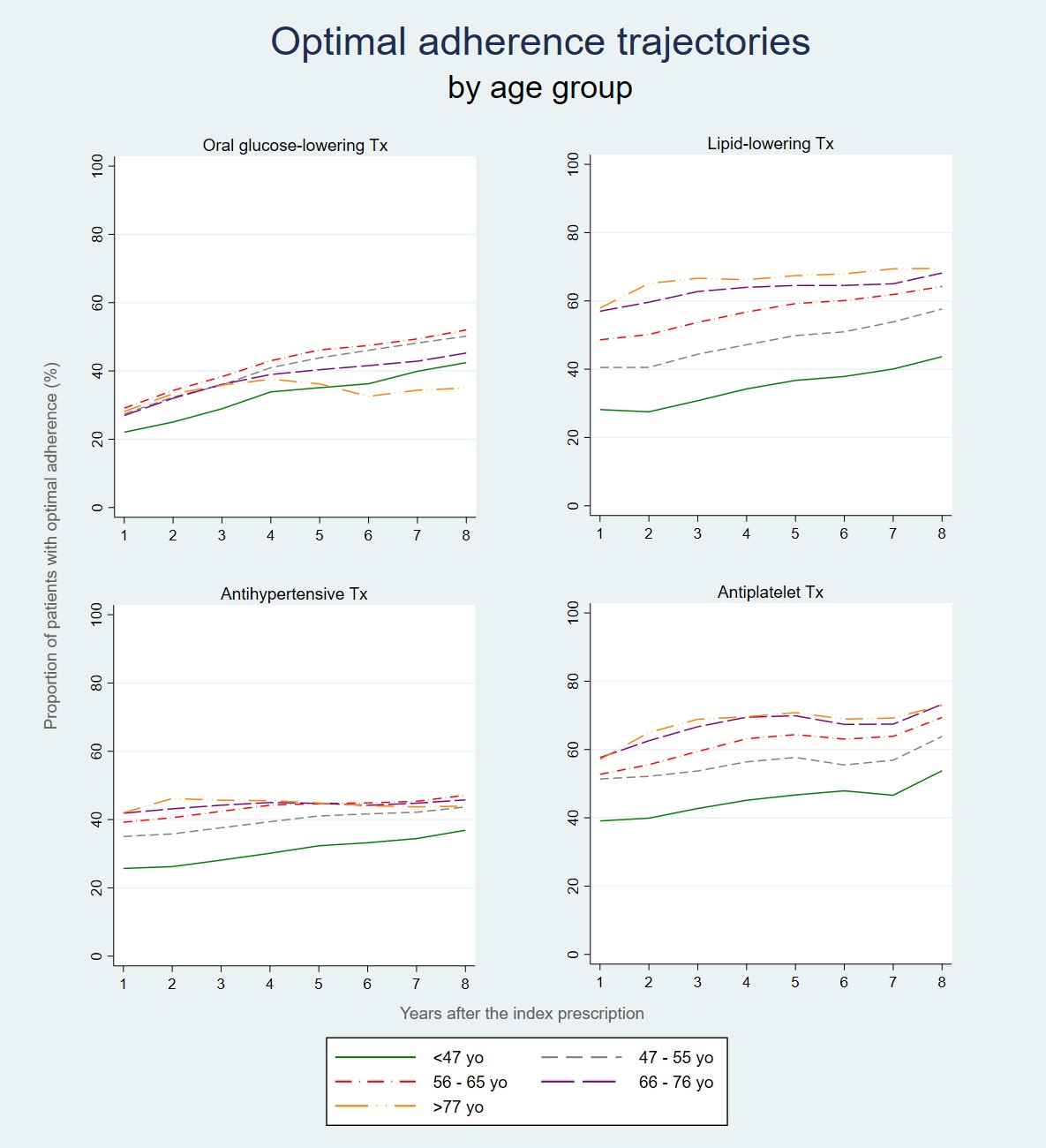


yo: years old; Tx: treatment

Optimal adherence is defined as an annual proportion of days covered ≥ 80%.


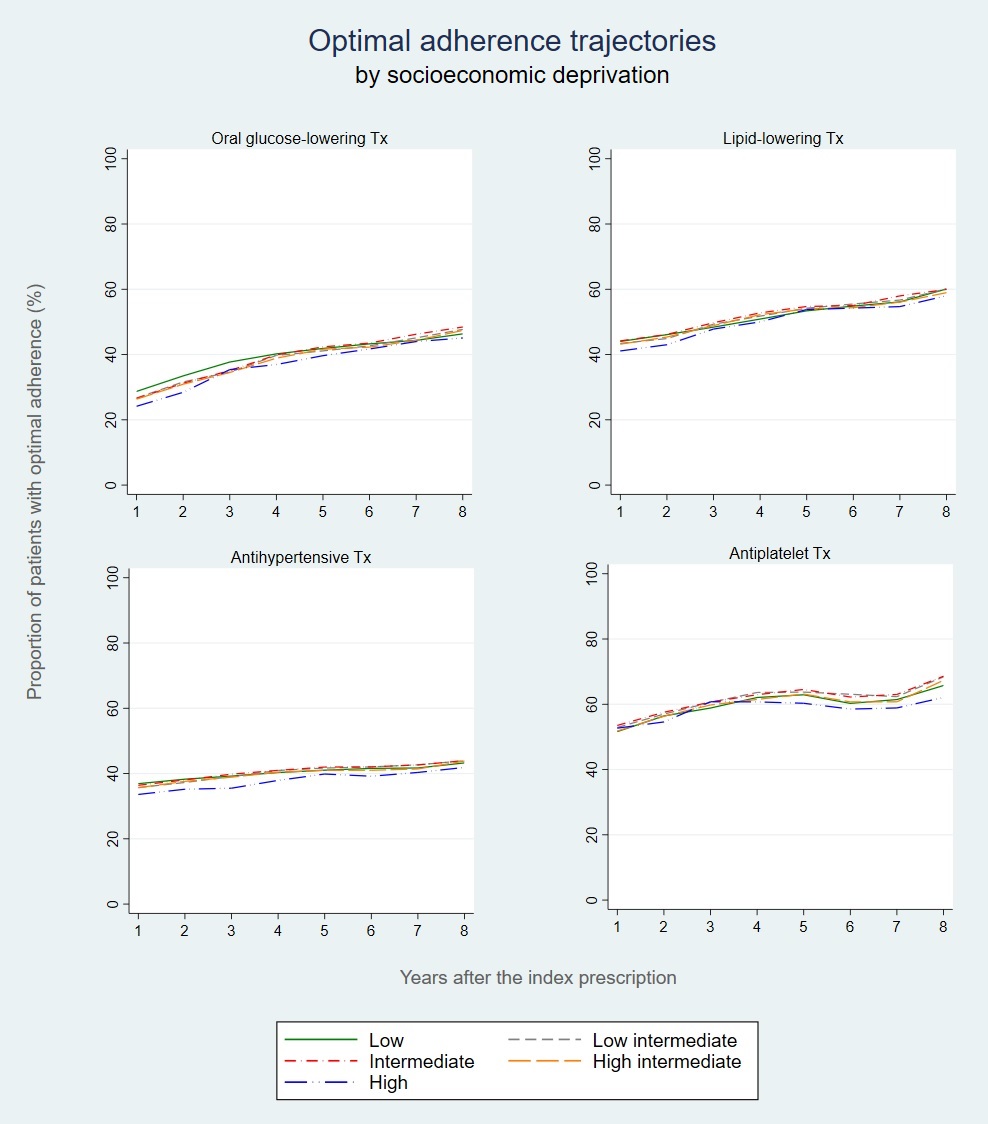


Tx: treatment

Optimal adherence is defined as an annual proportion of days covered ≥ 80%.

#
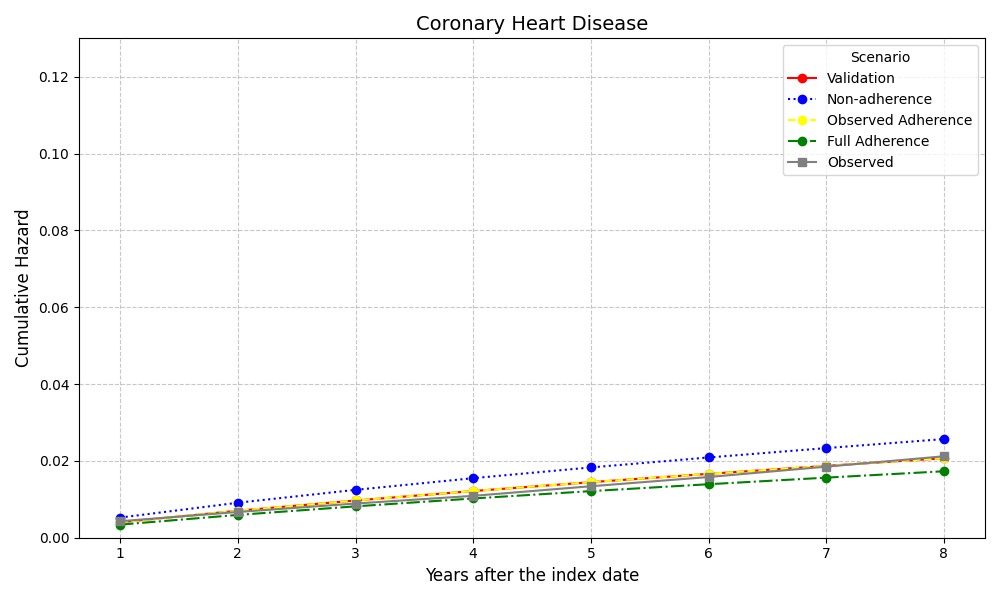
**Supplementary Figure SF2.** Annual cumulative hazard over 8 years of follow-up for cardiovascular endpoints: observed data (Nelson–Aalen estimator) versus predictions from the CVD Markov model.


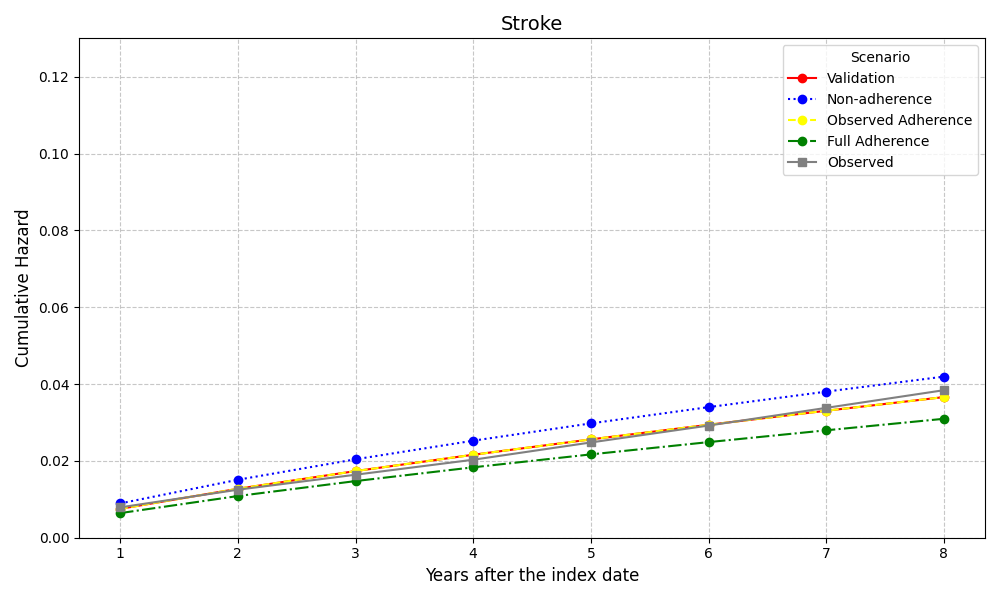


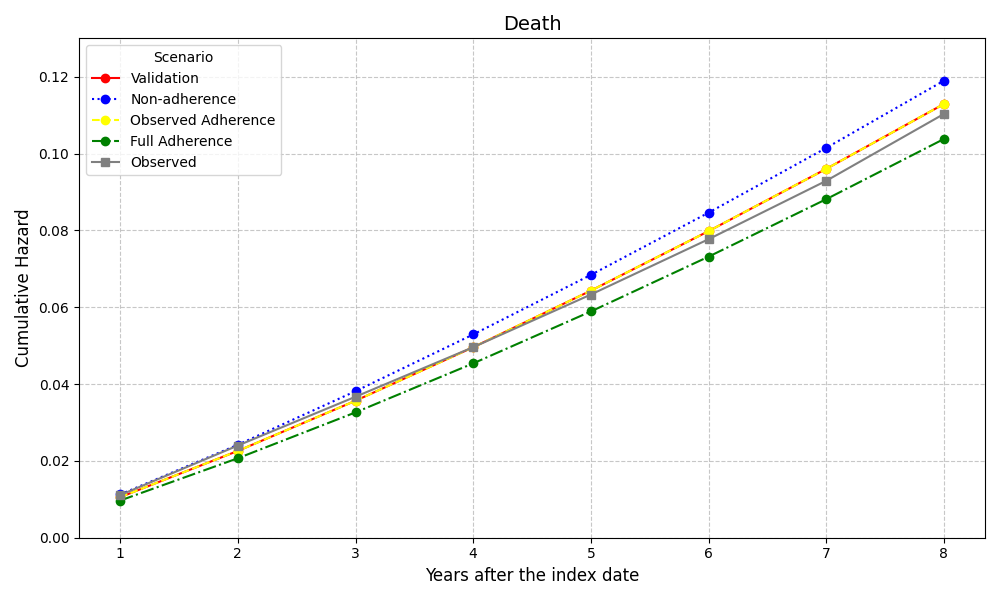

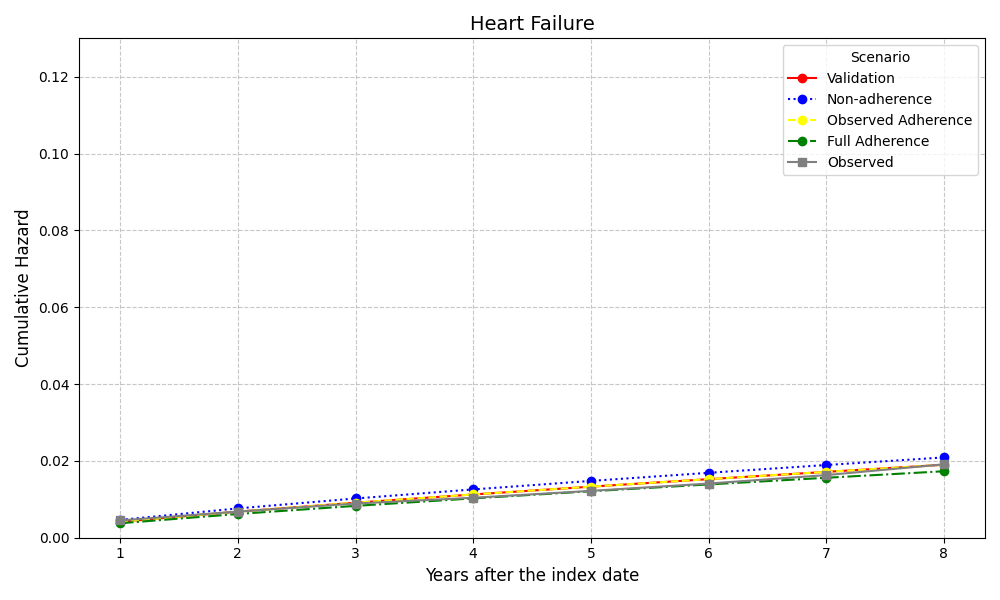


Note: Only the first event was used for the model development and validation when more than one event of the same category occurred for a patient in a given year since the index prescription.

# **Supplementary Figure SF3.** Mean predicted outcomes and costs over a lifetime, discounted at 3% across three scenarios of medication adherence by CVD prevention, sex and age, for antihypertensive, oral glucose-lowering, lipid-lowering, and antiplatelet medication.


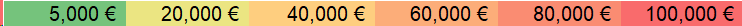

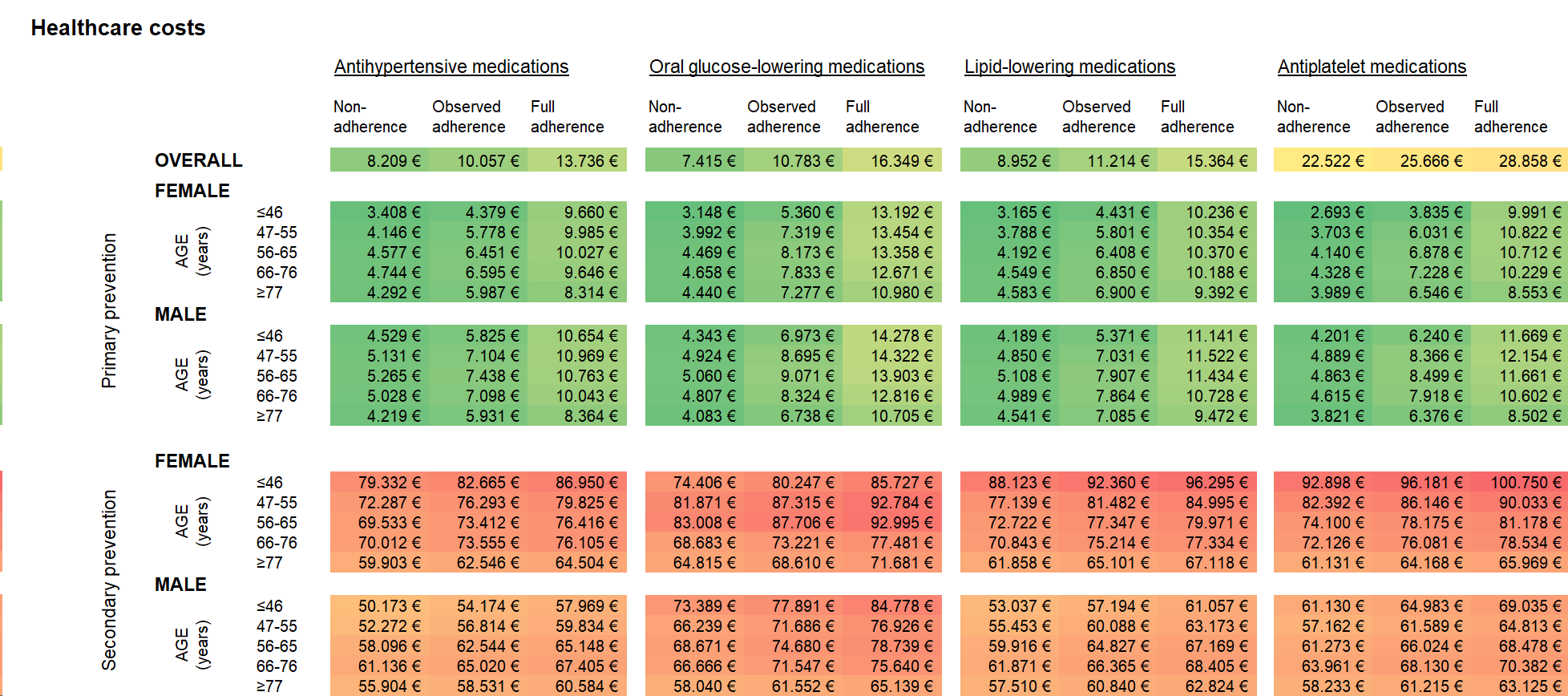

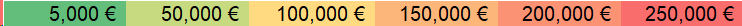

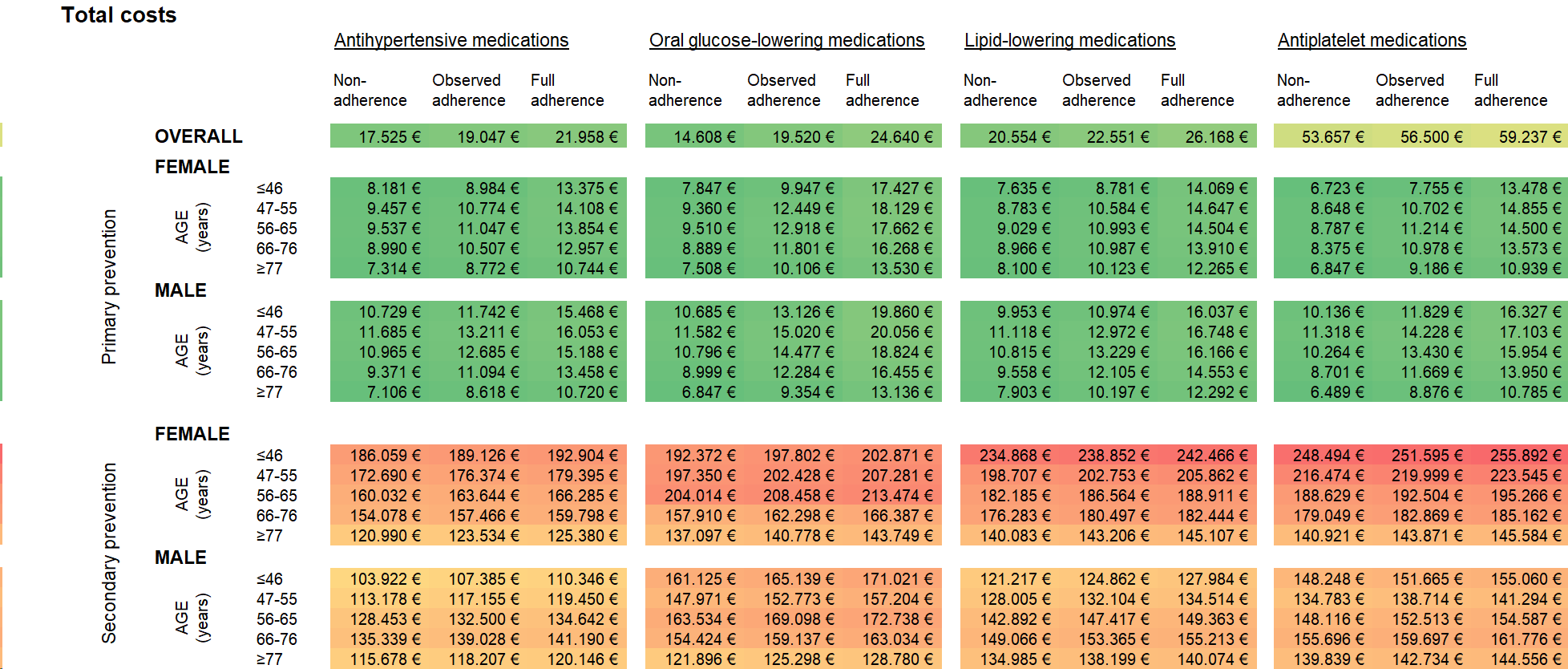


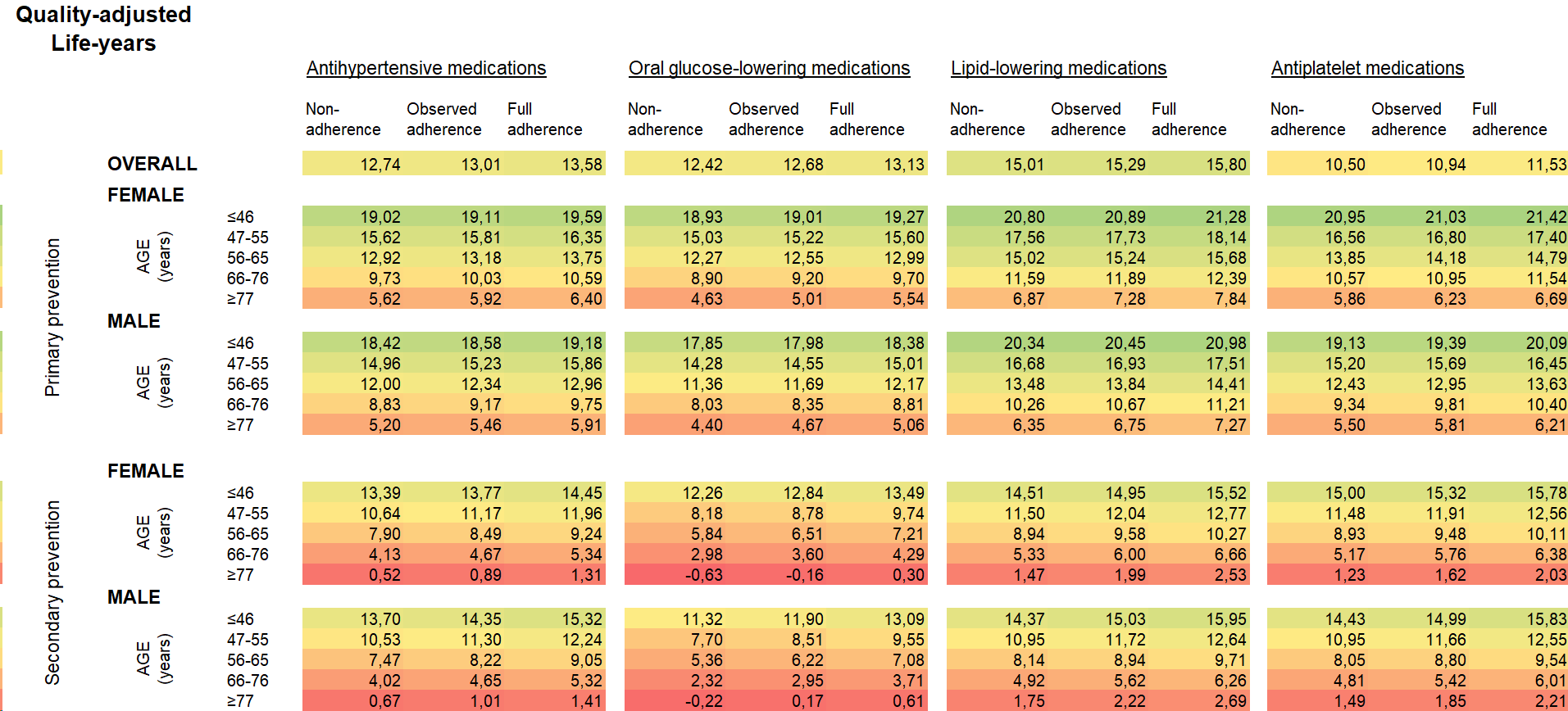

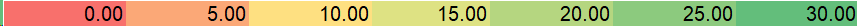


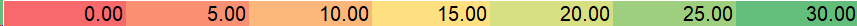

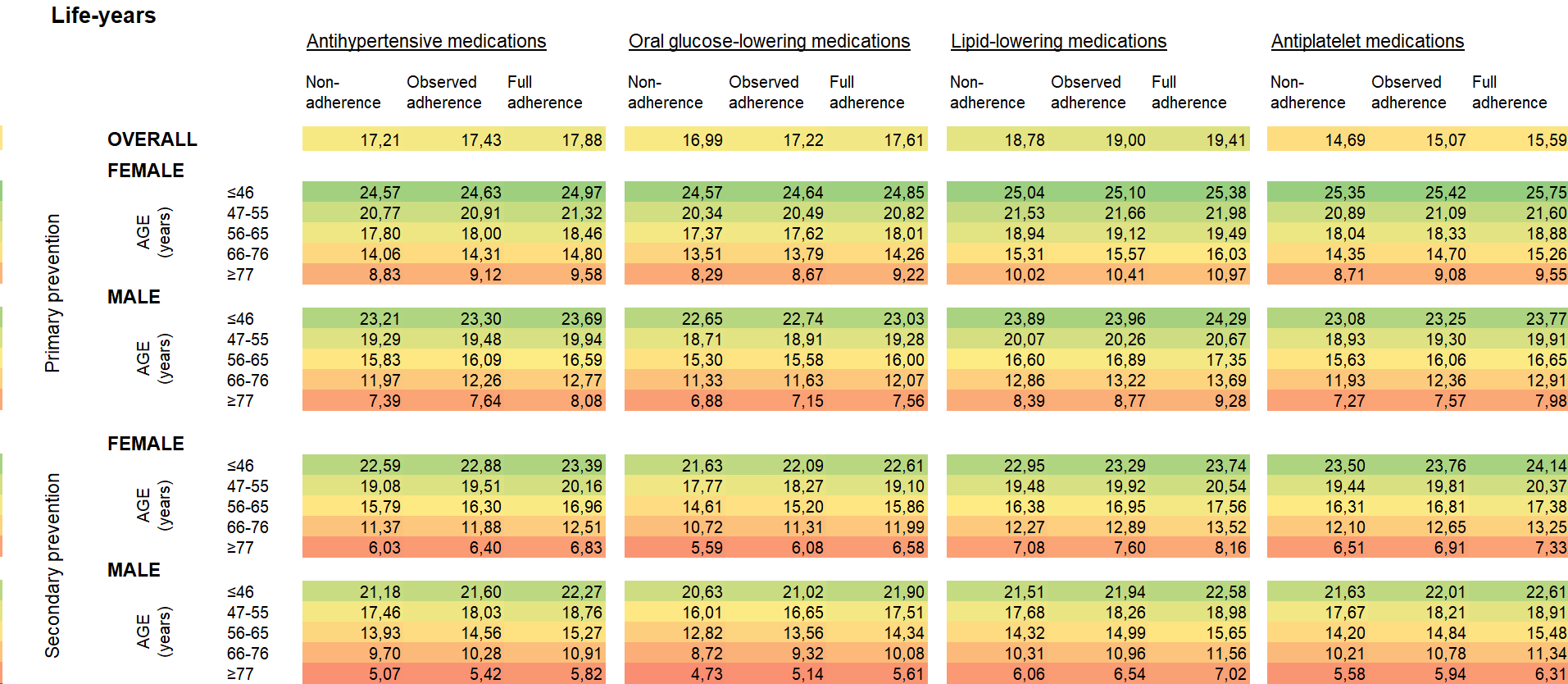


# **Supplementary Figure SF4.** Incremental cost per life year gained and incremental cost per QALY gained, discounted at 3% across three scenarios of medication adherence by CVD prevention, sex and age, for the overall cohort and by treatment group (antihypertensive, oral glucose-lowering, lipid-lowering, and antiplatelet medication).


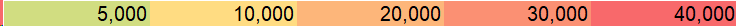

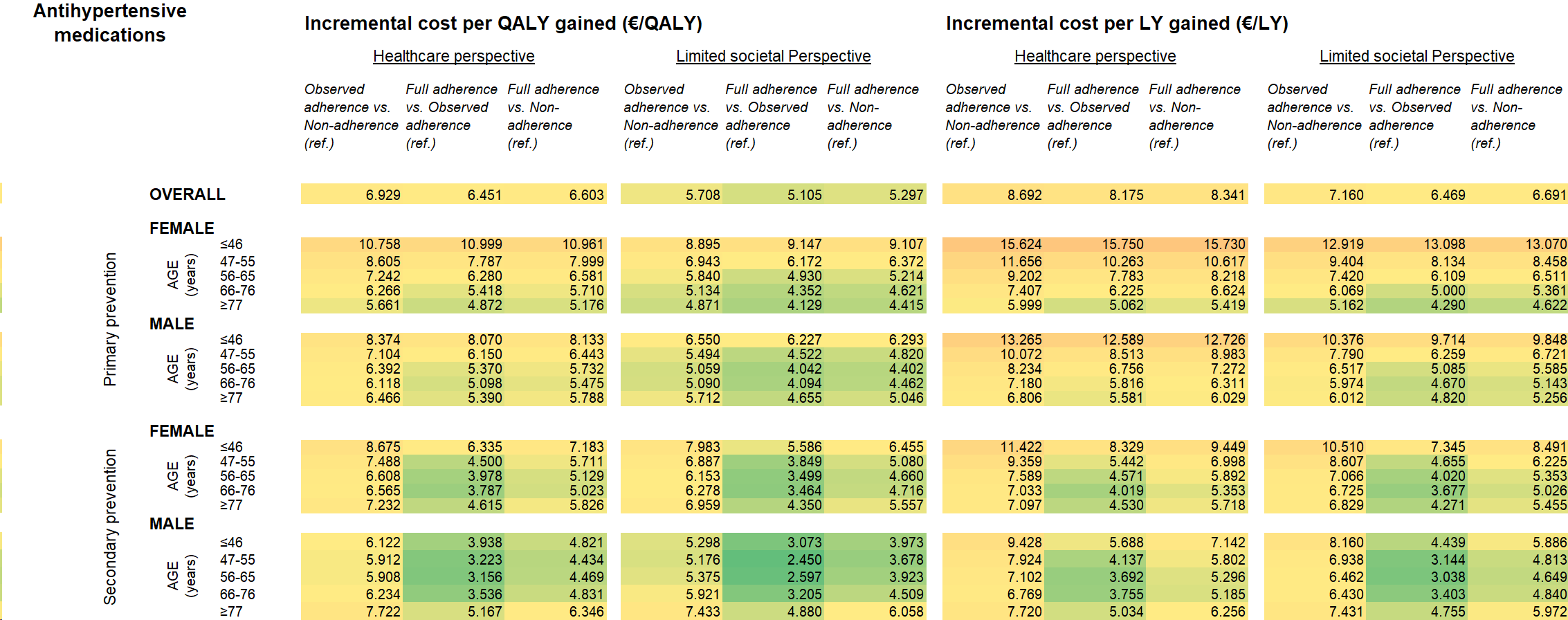

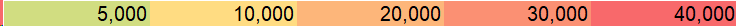

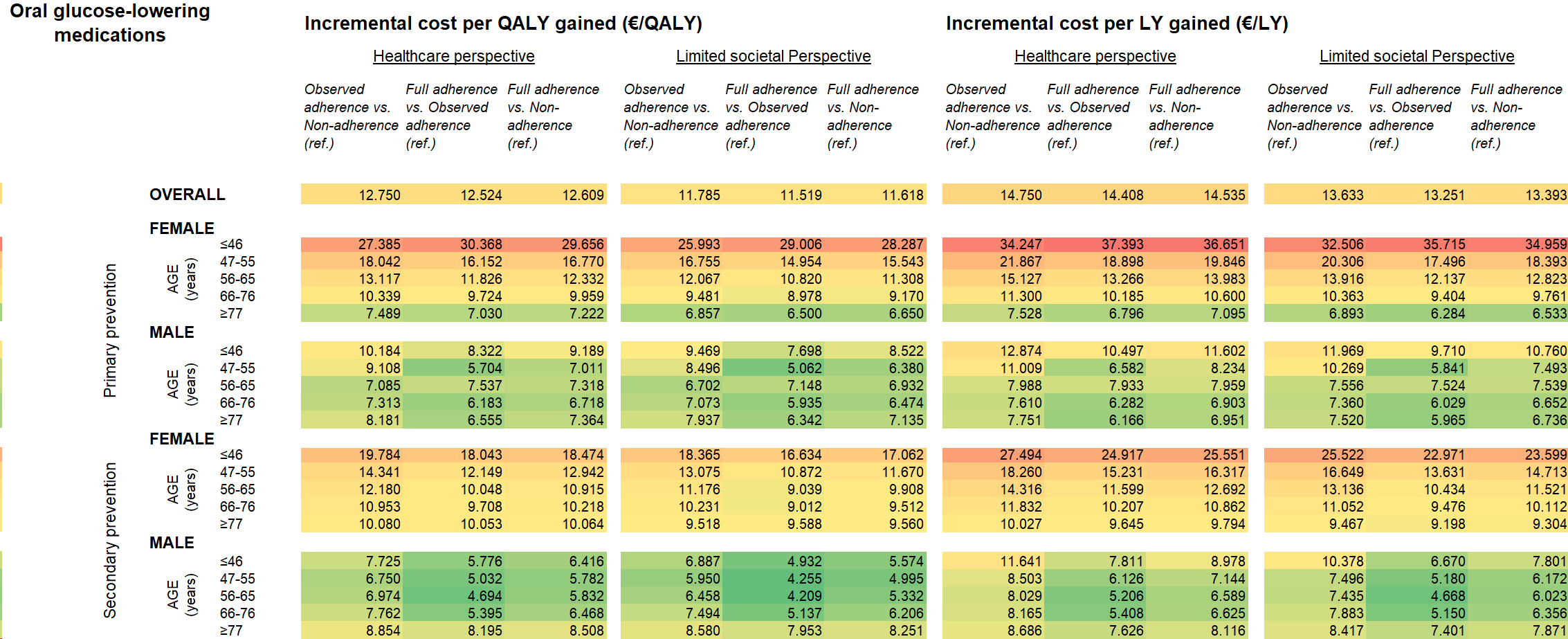

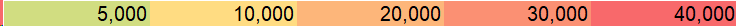

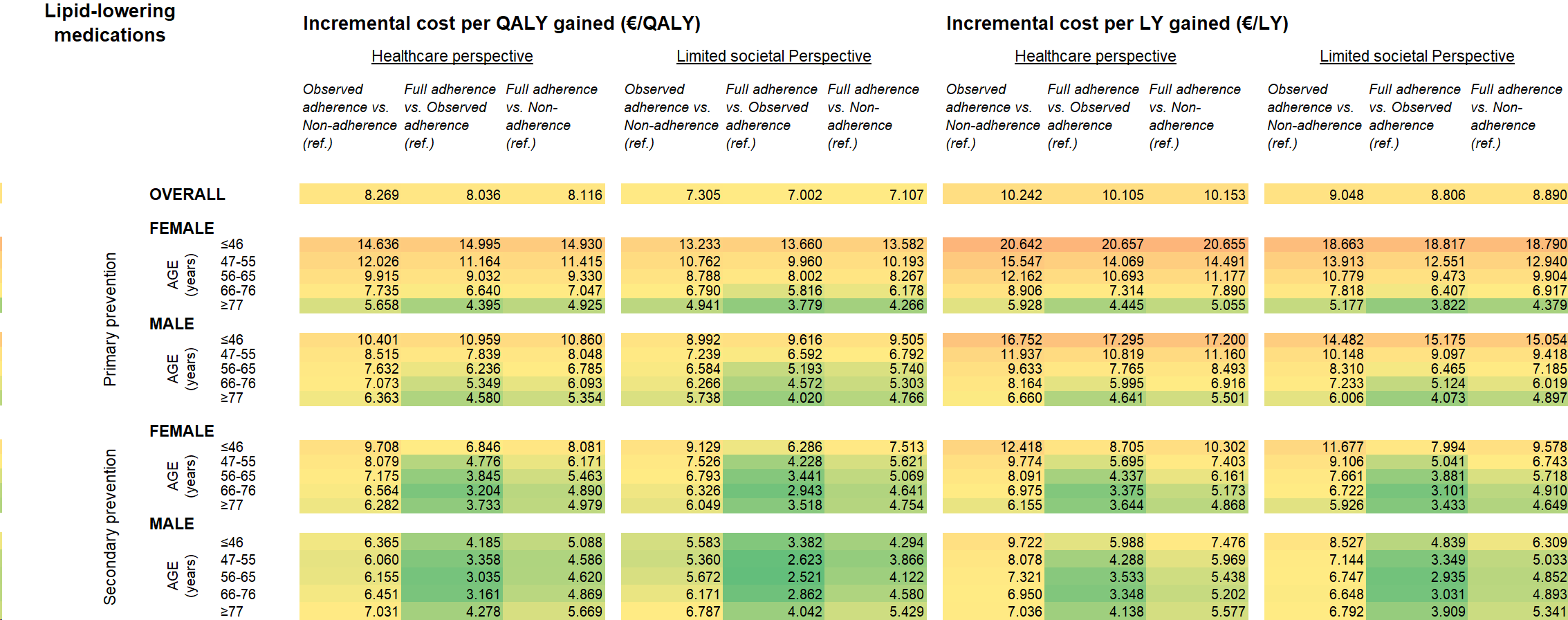

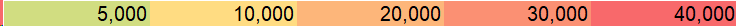

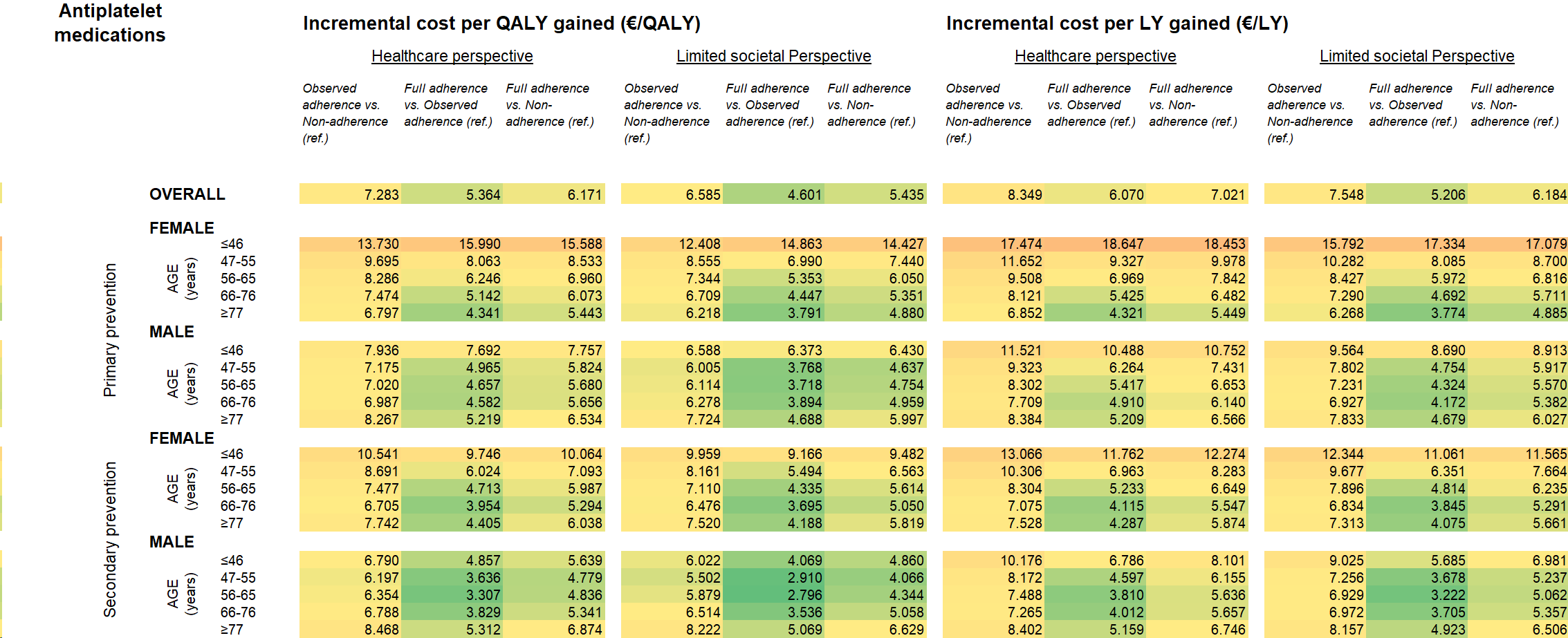


#
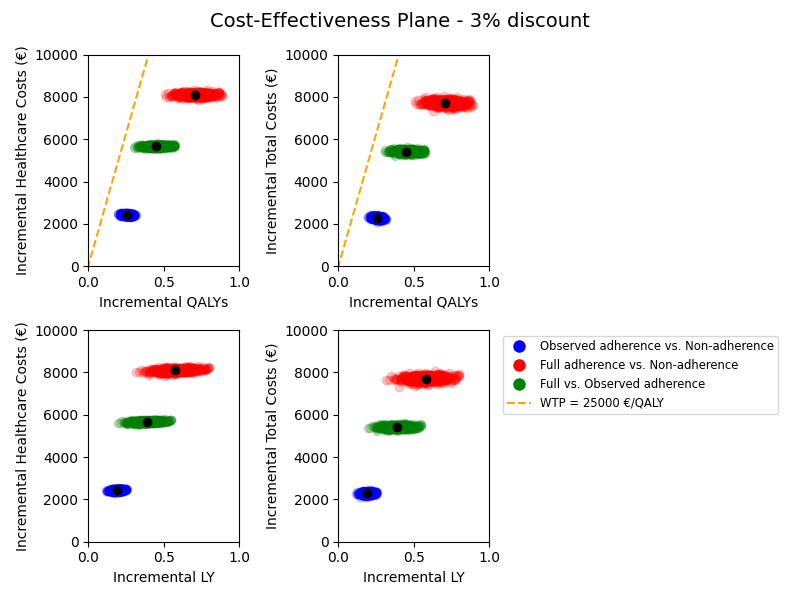
**Supplementary Figure SF5.** Cost-effectiveness planes showing results from the probabilistic sensitivity analysis (500 bootstrap replications) and deterministic analysis, with healthcare and societal costs plotted against QALYs and life-years.

The black dot represents the deterministic analysis.

#
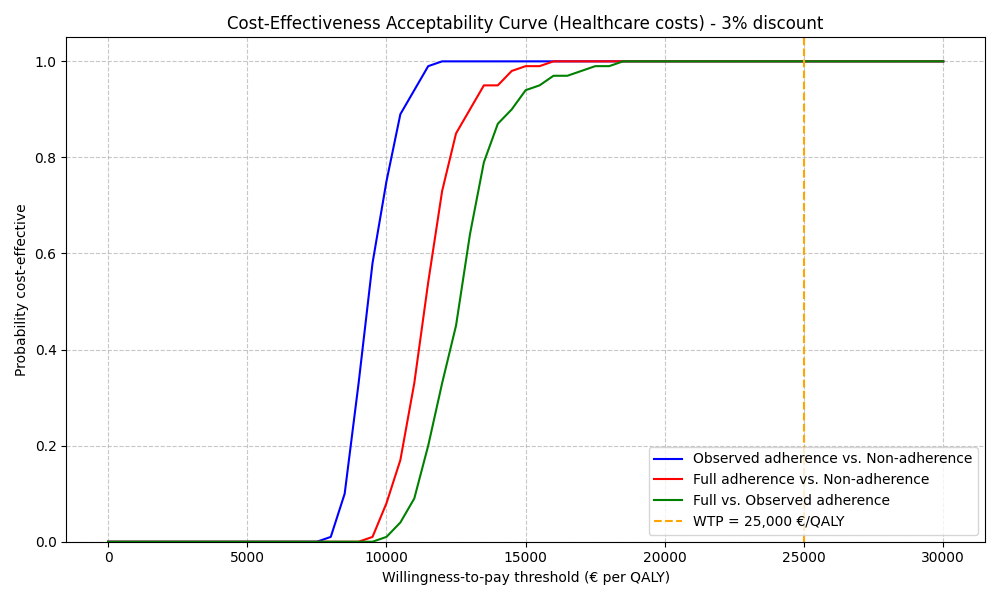
**Supplementary Figure SF6.** Cost-effectiveness acceptability curves for a willingness to pay threshold of 25.000€/QALY from the A) healthcare and B) societal perspectives.


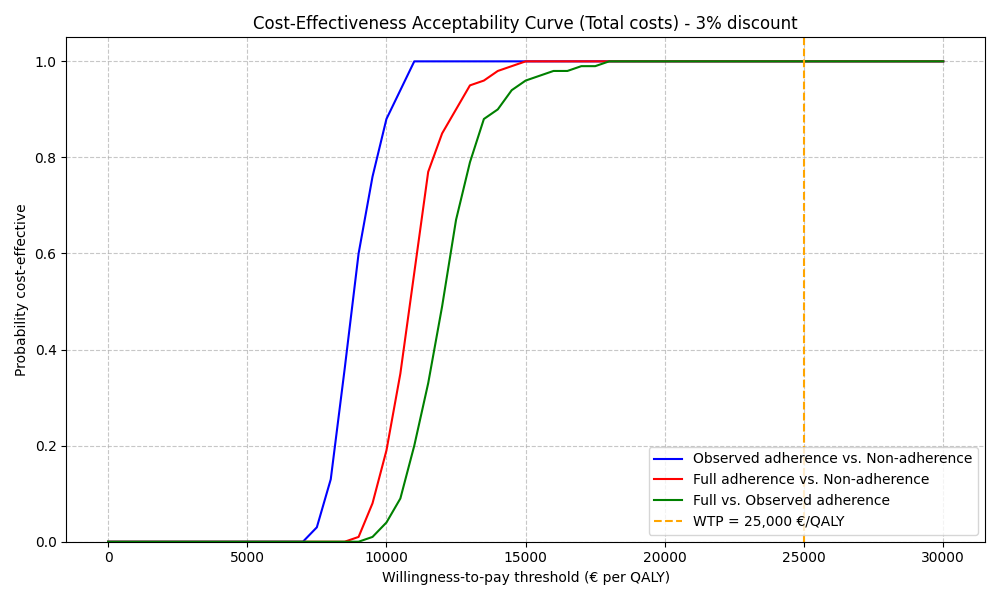


# **
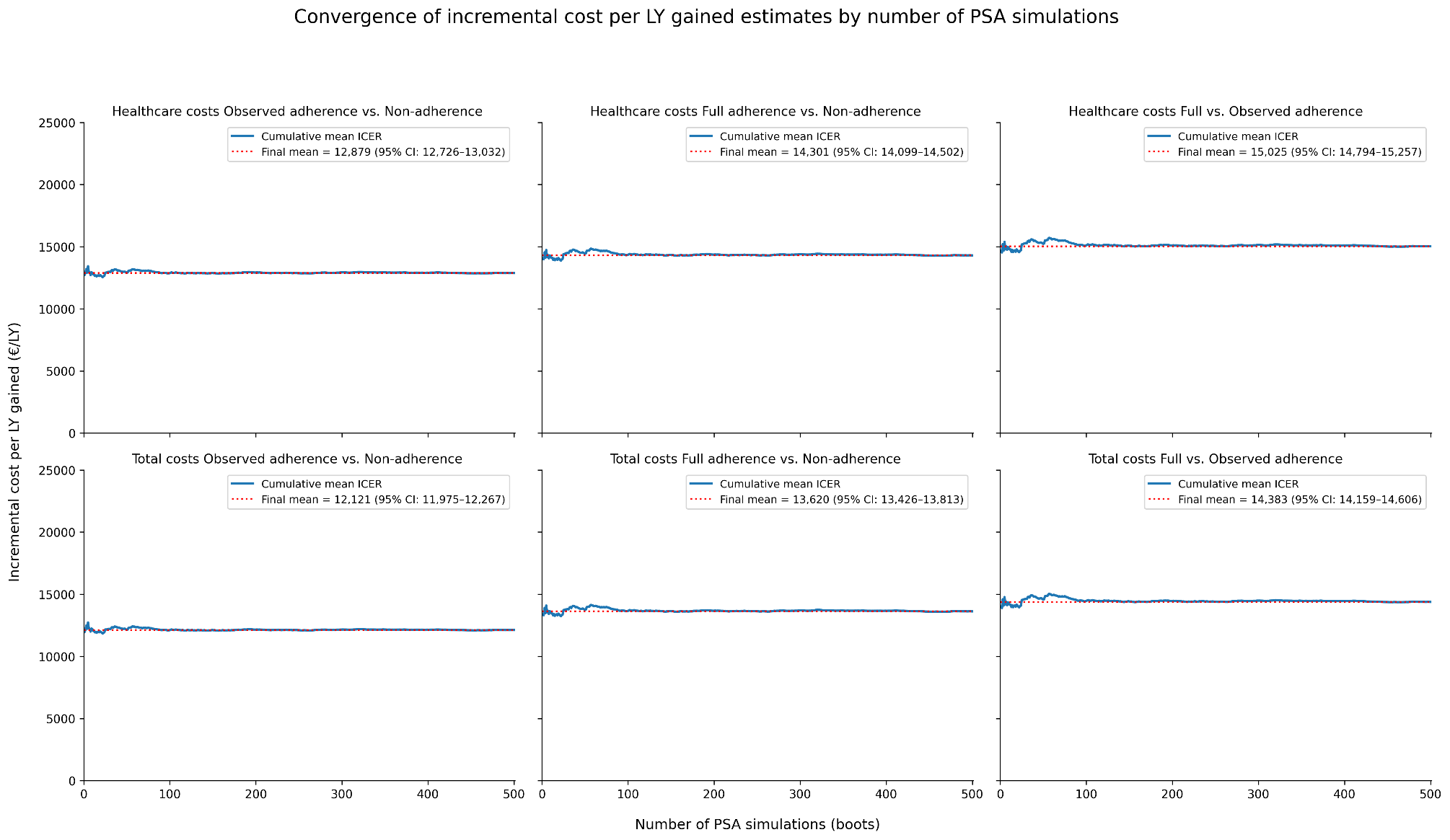
Supplementary Figure SF7.** Convergence of incremental cost per LY and QALY gained estimates across probabilistic sensitivity analysis simulations.

**
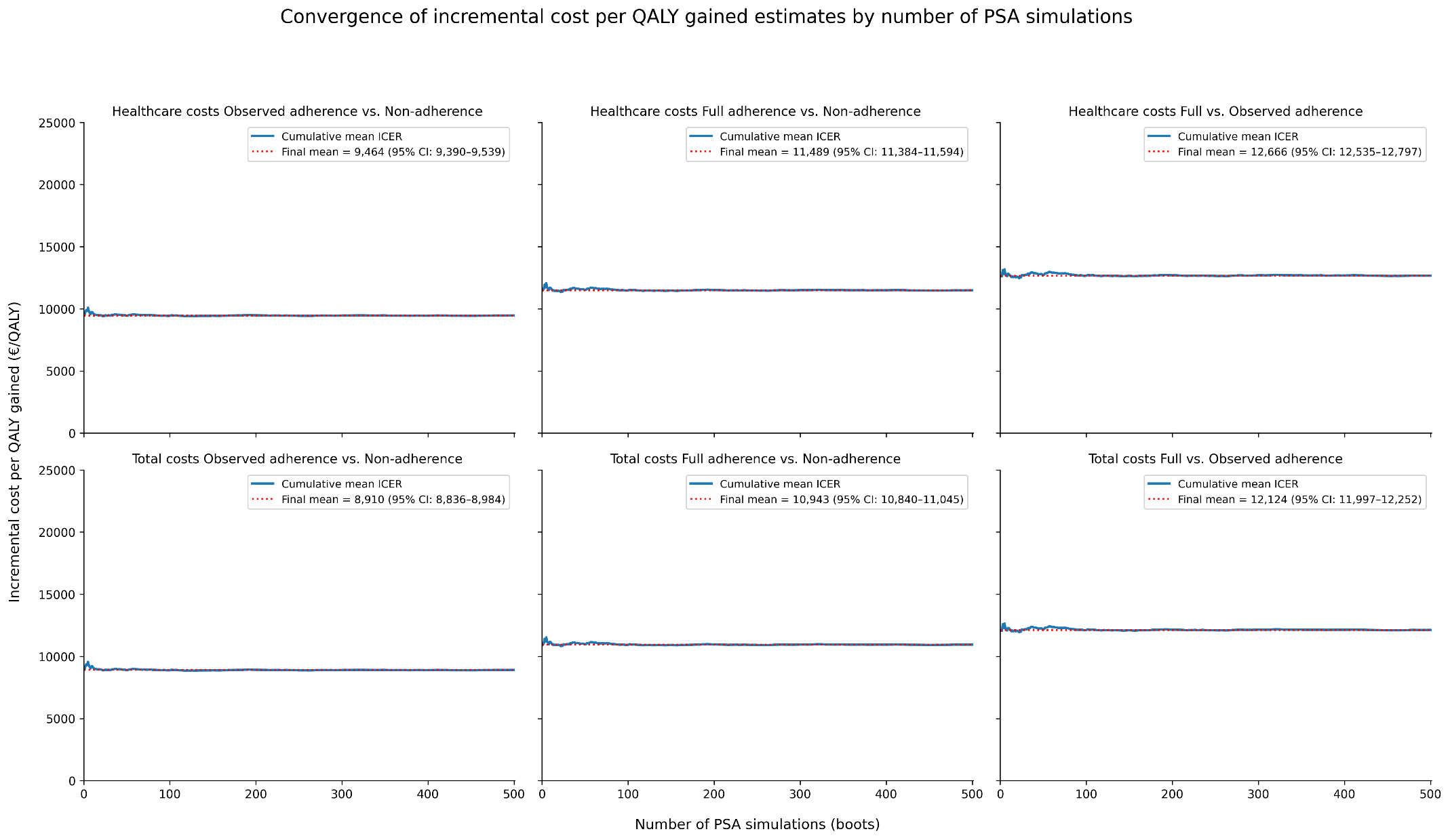
**

# **CHEERS 2022 Checklist**. Adapted from Husereau et al. [24]

| **Topic** | **No.** | **Item** | **Location where item is reported** |
| --- | --- | --- | --- |
| **Title** |  |  |  |
|  | 1 | Identify the study as an economic evaluation and specify the interventions being compared. | 1 |
| **Abstract** |  |  |  |
|  | 2 | Provide a structured summary that highlights context, key methods, results, and alternative analyses. | 3 |
| **Introduction** |  |  |  |
| **Background and objectives** | 3 | Give the context for the study, the study question, and its practical relevance for decision making in policy or practice. | 4-5 |
| **Methods** |  |  |  |
| **Health economic analysis plan** | 4 | Indicate whether a health economic analysis plan was developed and where available. | NA |
| **Study population** | 5 | Describe characteristics of the study population (such as age range, demographics, socioeconomic, or clinical characteristics). | 5 |
| **Setting and location** | 6 | Provide relevant contextual information that may influence findings. | 5 |
| **Comparators** | 7 | Describe the interventions or strategies being compared and why chosen. | 7 |
| **Perspective** | 8 | State the perspective(s) adopted by the study and why chosen. | 8-9 |
| **Time horizon** | 9 | State the time horizon for the study and why appropriate. | 6 |
| **Discount rate** | 10 | Report the discount rate(s) and reason chosen. | 6 |
| **Selection of outcomes** | 11 | Describe what outcomes were used as the measure(s) of benefit(s) and harm(s). | 6 |
| **Measurement of outcomes** | 12 | Describe how outcomes used to capture benefit(s) and harm(s) were measured. | 7-8 |
| **Valuation of outcomes** | 13 | Describe the population and methods used to measure and value outcomes. | 7-8 |
| **Measurement and valuation of resources and costs** | 14 | Describe how costs were valued. | 8-9 |
| **Currency, price date, and conversion** | 15 | Report the dates of the estimated resource quantities and unit costs, plus the currency and year of conversion. | 8 |
| **Rationale and description of model** | 16 | If modelling is used, describe in detail and why used. Report if the model is publicly available and where it can be accessed. | 6 |
| **Analytics and assumptions** | 17 | Describe any methods for analysing or statistically transforming data, any extrapolation methods, and approaches for validating any model used. | 8 |
| **Characterising heterogeneity** | 18 | Describe any methods used for estimating how the results of the study vary for subgroups. | 9 |
| **Characterising distributional effects** | 19 | Describe how impacts are distributed across different individuals or adjustments made to reflect priority populations. | NA |
| **Characterising uncertainty** | 20 | Describe methods to characterise any sources of uncertainty in the analysis. | 9-10 |
| **Approach to engagement with patients and others affected by the study** | 21 | Describe any approaches to engage patients or service recipients, the general public, communities, or stakeholders (such as clinicians or payers) in the design of the study. | NA |
| **Results** |  |  |  |
| **Study parameters** | 22 | Report all analytic inputs (such as values, ranges, references) including uncertainty or distributional assumptions. | 10 |
| **Summary of main results** | 23 | Report the mean values for the main categories of costs and outcomes of interest and summarise them in the most appropriate overall measure. | 11-12 |
| **Effect of uncertainty** | 24 | Describe how uncertainty about analytic judgments, inputs, or projections affect findings. Report the effect of choice of discount rate and time horizon, if applicable. | 12 |
| **Effect of engagement with patients and others affected by the study** | 25 | Report on any difference patient/service recipient, general public, community, or stakeholder involvement made to the approach or findings of the study | NA |
| **Discussion** |  |  |  |
| **Study findings, limitations, generalisability, and current knowledge** | 26 | Report key findings, limitations, ethical or equity considerations not captured, and how these could affect patients, policy, or practice. | 12-14 |
| **Other relevant information** |  |  |  |
| **Source of funding** | 27 | Describe how the study was funded and any role of the funder in the identification, design, conduct, and reporting of the analysis | 15 |
| **Conflicts of interest** | 28 | Report authors conflicts of interest according to journal or International Committee of Medical Journal Editors requirements. | 15 |

**REFERENCES**

1. Duque I, Domínguez-Berjón MF, Cebrecos A, Prieto-Salceda MD, Esnaola S, Calvo Sánchez M, et al. Índice de privación en España por sección censal en 2011. Gaceta Sanitaria. 2021;35:113–22. https://doi.org/10.1016/j.gaceta.2019.10.008

2. Turnbull F, Neal B, Algert C, Chalmers J, Woodward M, MacMahon S, et al. Effects of different blood-pressure-lowering regimens on major cardiovascular events: results of prospectively-designed overviews of randomised trials. The Lancet. Elsevier Limited; 2003;362:1527–35. https://doi.org/10.1016/S0140-6736(03)14739-3

3. Ghosh-Swaby OR, Goodman SG, Leiter LA, Cheng A, Connelly KA, Fitchett D, et al. Glucose-lowering drugs or strategies, atherosclerotic cardiovascular events, and heart failure in people with or at risk of type 2 diabetes: an updated systematic review and meta-analysis of randomised cardiovascular outcome trials. Lancet Diabetes Endocrinol. Lancet Diabetes Endocrinol; 2020;8:418–35. https://doi.org/10.1016/S2213-8587(20)30038-3

4. Baigent C, Blackwell L, Emberson J, Holland LE, Reith C, Bhala N, et al. Efficacy and safety of more intensive lowering of LDL cholesterol: A meta-analysis of data from 170 000 participants in 26 randomised trials. The Lancet. Elsevier B.V.; 2010;376:1670–81. https://doi.org/10.1016/S0140-6736(10)61350-5

5. Preiss D, Campbell RT, Murray HM, Ford I, Packard CJ, Sattar N, et al. The effect of statin therapy on heart failure events: a collaborative meta-analysis of unpublished data from major randomized trials. Eur Heart J. 2015;36:1536–46. https://doi.org/10.1093/eurheartj/ehv072

6. Collins R, Peto R, Hennekens C, Doll R, Bubes V, Buring J, et al. Aspirin in the primary and secondary prevention of vascular disease: collaborative meta-analysis of individual participant data from randomised trials. The Lancet. Elsevier Ltd; 2009;373:1849–60. https://doi.org/10.1016/S0140-6736(09)60503-1

7. de Oliveira ABM, Luchiari B, Bonilha I, Barreto J, Nogueira ACC, Ceniccola GD, et al. Aspirin in primary prevention and the risk of heart failure: a systematic review and meta‐analysis of controlled trials. ESC Heart Fail. 2023;10:1488–91. https://doi.org/10.1002/ehf2.14269

8. Rubio R, Palacios B, Varela L, Fernández R, Camargo Correa S, Estupiñan MF, et al. Quality of life and disease experience in patients with heart failure with reduced ejection fraction in Spain: a mixed-methods study. BMJ Open. BMJ Open; 2021;11:e053216. https://doi.org/10.1136/bmjopen-2021-053216

9. Coll de Tuero G, Dalfó i Baqué A, de la Figuera Von Wichmann M, Gibert i Llorach E, Isnard Blanchar M, Martínez Alonso V, et al. Hipertensió Arterial. Institut Català del Salut. Barcelona; 2012;Guies de p.

10. Mata M, Cos FX, Morros R, Diego L, Barrot J, Berengué M, et al. Abordatge de la diabetis mellitus tipus 2. Guies de pràctica clínica ICS: Dislipemia. 2015;Guies de p.

11. Franzi Sisó A, Armengol Alegre J, Baena Díez JM, Barceló Colomer E, Ciurana Misol R, Esteve Lafuente E, et al. Guia de lípids i risc cardiovascular. Institut Català de la Salut. Barcelona; 2021;

12. Departament de Salut. RESOLUCIÓ SLT/4326/2023, de 20 de desembre, sobre la revisió de preus públics corresponents als serveis sanitaris que presta l’Institut Català de la Salut. Diari Oficial de la Generalitat de Catalunya. 2023;9067:1–199.

13. Ministerio de Sanidad. Información sobre los productos incluidos en la prestación farmacéutica del SNS (dispensables a través de oficinas de farmacia) [Internet]. 2024. https://www.sanidad.gob.es/profesionales/nomenclator.do

14. Darbà J, Marsà A. Burden of ischemic heart disease in Spain: incidence, hospital mortality and costs of hospital care. Expert Rev Pharmacoecon Outcomes Res. 2022;22:1147–52. https://doi.org/10.1080/14737167.2022.2108794

15. Soler-Font M, Ribera A, Aznar-Lou I, Sánchez-Viñas A, Slof J, Vela E, et al. Health and social costs during the first year after stroke by degree of functional disability. International Journal of stroke. European Journal of Stroke. 2024;

16. Leal J, Luengo-Fernández R, Gray A, Petersen S, Rayner M. Economic burden of cardiovascular diseases in the enlarged European Union. Eur Heart J. 2006;27:1610–9. https://doi.org/10.1093/eurheartj/ehi733

17. Escobar C, Palacios B, Varela L, Gutiérrez M, Duong M, Chen H, et al. Healthcare resource utilization and costs among patients with heart failure with preserved, mildly reduced, and reduced ejection fraction in Spain. BMC Health Serv Res. 2022;22:1241. https://doi.org/10.1186/s12913-022-08614-x

18. Instituto Nacional de Estadística. Tasas de actividad, paro y empleo por provincia y sexo [Internet]. 2023 [cited 2024 Feb 5]. https://www.ine.es/jaxiT3/Datos.htm?t=3996. Accessed 5 Feb 2024

19. Instituto Nacional de Estadística. Salarios medios [Internet]. 2022 [cited 2024 Feb 5]. https://www.ine.es/jaxiT3/Datos.htm?t=13926. Accessed 5 Feb 2024

20. Ministerio de Inclusión Seguridad Social y Migraciones. Requisitos Jubilación Ordinaria [Internet]. 2024. https://www.seg-social.es/wps/portal/wss/internet/Trabajadores/PrestacionesPensionesTrabajadores/10963/28393/28396/28472

21. WHO Collaborating Centre for Drug Statistics Methodology, Norwegian Institute of Public Health. ATC/DDD Index 2024 [Internet]. 2024 [cited 2024 Aug 15]. https://atcddd.fhi.no/atc_ddd_index/. Accessed 15 Aug 2024

22. World Health Organization. International Classification of Diseases Tenth Revision (ICD-10). Sixth Edit. Geneva; 2019.

23. Dalli LL, Kilkenny MF, Arnet I, Sanfilippo FM, Cummings DM, Kapral MK, et al. Towards better reporting of the proportion of days covered method in cardiovascular medication adherence: A scoping review and new tool TEN‐SPIDERS. Br J Clin Pharmacol [Internet]. John Wiley & Sons, Ltd; 2022 [cited 2025 Sep 11];88:4427–42. https://doi.org/10.1111/bcp.15391

24. Husereau D, Drummond M, Augustovski F, de Bekker-Grob E, Briggs AH, Carswell C, et al. Consolidated Health Economic Evaluation Reporting Standards 2022 (CHEERS 2022) statement: updated reporting guidance for health economic evaluations. The European Journal of Health Economics [Internet]. Eur J Health Econ; 2022 [cited 2022 May 16]; https://doi.org/10.1007/s10198-021-01426-6
